# Reduction of stress responses in honey bees by synthetic ligands targeting an allatostatin receptor

**DOI:** 10.1038/s41598-022-20978-y

**Published:** 2022-10-06

**Authors:** Adrià Sánchez-Morales, Véronique Gigoux, Minos-Timotheos Matsoukas, Laura Perez-Benito, Daniel Fourmy, Ramón Alibes, Félix Busqué, Arnau Cordomí, Jean-Marc Devaud

**Affiliations:** 1 Centre de Recherches Sur La Cognition Animale (CRCA), Centre de Biologie Intégrative, 9 (CBI), Université de Toulouse, CNRS, Toulouse, France; 2 Departament de Química, Universitat Autònoma de Barcelona, Bellaterra, 08193 Barcelona, Spain; 3 Centre de Recherches en Cancérologie de Toulouse (CRCT), INSERM Unité Mixte de Recherche UMR-1037, CNRS Equipe de Recherche Labellisée ERL5294, Université de Toulouse, Toulouse, France; 4 Unitat de Bioestadística, Universitat Autònoma de Barcelona, Bellaterra, 08193 Barcelona, Spain; 5 Present Address: Bioinformatics, ESCI-Universitat Pompeu Fabra, Passeig Pujades 1, 08003 Barcelona, Spain; 6 Present Address: Computational Chemistry, Janssen Research & Development, Janssen Pharmaceutica N. V., Turnhoutseweg 30, B-2340 Beerse, Belgium

**Keywords:** Virtual screening, Classical conditioning, Chemical synthesis, Feeding behaviour

## Abstract

Honey bees are of great economic and ecological importance, but are facing multiple stressors that can jeopardize their pollination efficiency and survival. Therefore, understanding the physiological bases of their stress response may help defining treatments to improve their resilience. We took an original approach to design molecules with this objective. We took advantage of the previous identified neuropeptide allatostatin A (ASTA) and its receptor (ASTA-R) as likely mediators of the honey bee response to a biologically relevant stressor, exposure to an alarm pheromone compound. A first series of ASTA-R ligands were identified through in silico screening using a homology 3D model of the receptor and in vitro binding experiments. One of these (A8) proved also efficient in vivo, as it could counteract two behavioral effects of pheromone exposure, albeit only in the millimolar range. This putative antagonist was used as a template for the chemical synthesis of a second generation of potential ligands. Among these, two compounds showed improved efficiency in vivo (in the micromolar range) as compared to A8 despite no major improvement in their affinity for the receptor in vitro. These new ligands are thus promising candidates for alleviating stress in honey bees.

## Introduction

Bees are vital to the survival of our ecosystem, by pollinating our crops, flowers and up to a third of all the food we eat^1^. The number of wild bees has been on decline in recent years, and population losses observed by many beekeepers are due to exposure to multiple stressors^2^. Thus, understanding physiological stress responses in this species, and identifying possible strategies to modulate them, is expected to open perspectives to improve their resilience^2,3^.

Stress responses allow individuals to cope with adverse situations or stimuli through adapted physiological and behavioral outcomes, and are mediated by various neural and hormonal signals^4^. Although such responses have been mostly studied in mammals, a few studies have contributed to better characterizing potential stress signals in bees and other model invertebrate species. Among such signals are neuropeptides^3,5–8^, a class of ubiquitous neuromodulators mediating neuroadaptive control of many organisms’ internal state^7–10^. As such, various neuropeptide receptors have been identified as potential therapeutic targets in humans^10–12^, but might also be interesting for developing strategies to reduce stress and improve welfare in production animals.

A natural stressor to honey bees is the sting alarm pheromone (SAP), a complex mixture of odors released by bees facing a threat, and inducing the recruitment of nestmates to defend the colony^13^. In addition to increasing aggressiveness, SAP and its main component isopentylacetate (IPA) induce other behavioral and physiological responses which remind strongly of the vertebrate stress response: increased respiratory rate, reduced sensitivity to noxious stimuli (‘analgesia’), decreased motivation for food, and learning impairment^6,14–18^. Allatostatins are a family of invertebrate neuropeptides, three of which were identified in adult brain of honey bees (ASTA, ASTC, ASTCC), as well as their respective receptor. While the C receptor was shown to be targeted by two ligands (ASTC and ASTC CC), the A receptor is specific for ASTA, and shares sequence similarities with opioid and galanin receptors, its closest mammalian relatives^6^. Since injecting allatostatins in the micromolar range to unstressed bees mimics the learning impairment induced by IPA exposure, they can be considered as likely mediators of stress response^6,19^. In *Drosophila*, ASTA also acts as a satiety signal that modulates food intake and maintenance of nutrient homeostasis, through the inhibition of food- research behavior^20,21^. It participates into a more general action of promoting an energy-saving state through the coordination of feeding, digestion, locomotor activity and sleep, particularly under nutritional stress conditions^22^. Motivation for sugar is particularly sensitive to ASTA signaling: intake, acceptance, responsiveness and preference for this nutrient are reduced by ASTA^20,21^. In addition, brain ASTA-expressing neurons also modulate sucrose-mediated appetitive learning in fruitflies^23^. Interestingly, these biological functions are reminiscent of the capacity of both opioid and galanin signaling to modulate many aspects of stress response, including the reward system^24,25^, leading to search for receptor agonists or antagonists with protective effects against stress in rodent systems (e.g.^26–28^). Likewise, we asked whether synthetic ligands of allatostatin receptors could have similar actions. Here, we searched for ligands that might antagonize the activation of the A receptor by its ligand, ASTA, by combining in silico screening, binding assays and behavioral tests.

## Methods

### In silico screening of candidate molecules

A homology model of the honey bee allatostatin A receptor ASTA-R (NCBI: XP_006560262.1) was created using Modeller 9.12^29^ based on the crystal structure of the antagonist-bound human δ-opioid receptor (PDB id 4N6H)^30^. We constructed a pharmacophore model based on Electrostatic Maps (hydrophobic, hydrogen bond donor and hydrogen bond acceptor) as implemented in MOE 2013.08 (Molecular Operating Environment, Chemical Computing) which consisted in 13 pharmacophore features: 7 hydrophobic, 4 hydrogen bond acceptors and 2 hydrogen bond donors. Discovery Studio 3.5 software (Accelrys Software Inc., Discovery Studio Environment) was used for the virtual screening of ~ 5.5 million compounds from a filtered subset^31^ of the ZINC database^32^ using all pharmacophore models resulting from combinations of 5 to 7 pharmacophoric features out of the 13. To avoid steric clashes with the receptor, we used exclusion volumes derived from the receptor model. The fit value scoring was used to rank the compounds from best to worst fitting. Based on visual inspection, we selected 24 compounds from the best 1000 for experimental testing. Molecular dockings of the active compounds were performed with MOE 2016.08 using the Alpha Triangle method. The conformers were ranked by London dG scoring function to estimate the free energy of binding of the ligand from a given pose. Based on the dockings, we designed a scaffold for the next generation of ligands (‘B’ series).

### Binding assays

A subset of compounds selected from the screening were tested in a competition binding assay with ^125^I-allatostatin A transfected with allatostatin A receptor. HEK293T cells were grown onto 60-mm diameter culture dishes for 24 h in Dulbecco's Modified Eagle's medium (DMEM) supplemented with 10% of fetal bovine serum (FBS), in a humidified atmosphere at 95% air and 5% CO2. Cells were transfected using Lipofectamin 2000 (Invitrogen Life Technologies) following provider’s instructions (1 µg DNA/ 3 µl Lipofectamin 2000). 24 h later, cells were transferred to 24-well plates and incubated for 24 h. Cells were incubated for 60 min at 37 °C in 0.5 ml DMEM containing 0.1% bovine serum albumin with 5 nM of high performance liquid chromatography-purified Allatostatin-Dy647 (NHSDY647-PEG1, Dyomics GmbH, Jena, Germany) or ^125^I-Allatostatin in the presence or in the absence of increased concentrations of unlabeled antagonists or allatostatin. We used GRQPYSFGL-amide, the most abundant isoform of allatostatin A in the brain, which already proved effective both in vitro and in vivo^6^. Cells were washed twice with cold phosphate-buffered saline, pH 7.4, containing 2% bovine serum albumin and once with cold phosphate-buffered saline, pH 7.4. For binding studies using ^125^I-Allatostatin, cell-associated radioligand was collected by cell lysis with 0.1 N NaOH and the radioactivity was directly counted in a gamma counter (Auto-Gamma, Packard). For binding studies using allatostatin-Dy647, cells were recovered, transferred to flow cytometer tubes and cell-associated fluorescence was determined using a BD FACSCalibur™ flow cytometer. IC50 (concentration inhibiting half of specific binding) was calculated using the non-linear curve fitting software GraphPad Prism (San Diego, CA).

### Synthesis of second-generation ligands

#### General procedures

The solvents, chemicals, and reagents were acquired with high quality without any need for further purification from various commercial chemical companies such as, Merck (Darmstadt, Germany), Scharlab (Sentmenat, Spain), Apollo Scientific (Cheshire, United Kingdom), Alfa Aesar (kandel, Germany), and TCI (Zwijndrecht Belgium). Solvents were dried by distillation over the appropriate drying agents. All reactions were monitored by analytical thin-layer chromatography (TLC) using silica gel 60 precoated aluminum plates (0.20 mm thickness). Flash column chromatography was performed using silica gel Geduran® SI 60 (40–63 µm). ^1^H NMR and ^13^C NMR spectra were recorded at 250, 360, 400 MHz and 90, 100.6 MHz, respectively. ^19^F NMR spectra was recorded at 250 MHz (see Supplementary Material). Proton chemical shifts are reported in ppm (δ) (CDCl_3_: δ 7.26, acetone-d_6_: δ 2.05, DMSO-d_6_: δ 2.50, CD_2_Cl_2_: δ 5.32). Carbon chemical shifts are reported in ppm (δ) (CDCl_3_: δ 77.16, acetone-d_6_; δ 29.84, DMSO-d_6_: δ 39.52, CD_2_Cl_2_: δ 53.84). NMR signals were assigned with the help of HSQC, HMBC, DEPT135. Melting points were determined on hot stage and are uncorrected. HRMS were recorded using electrospray ionization (ESI).

#### (Benzylsulfanyl)acetic acid, 5a

A mixture of benzyl bromide (3.13 mL, 26.32 mmol), thioglycolic acid (1.84 mL, 26.32 mmol) in methanol (10 mL) was prepared. Then, a solution of sodium hydroxide (2.11 g, 52.64 mmol) in methanol (53 mL) was added slowly and the resulting mixture was stirred during 2 h. After this time, the solvent was removed in vacuum using a rotatory evaporator. The resulting white solid was dissolved in water (100 mL) and cleaned with AcOEt (2 × 75 mL). The aqueous layer was acidified dropwise with a solution of 1 M HCl until pH 3 and extracted with AcOEt (3 × 75 mL). The whole organic layers were rinsed with brine (100 mL). Finally, the solvent was evaporated under reduced pressure and the resulting solid was purified by hexane washings to furnish (benzylsulfanyl)acetic acid **5a** (2.87 g, 15.74 mmol, 60% yield) as a white solid. ^1^H NMR (360 MHz, DMSO-d_6_): δ 12.61 (s, 1H, -COO*H*), 7.35–7.24 (m, 5H, H-2^II^, H-3^II^, H-4^II^), 3.80 (s, 2H, H-2), 3.11 (s, 2H, H-1^I^). The spectroscopic data was consistent with the literature^33^.

#### (Phenethylsulfanyl)acetic acid, 5b

Compound 5b was prepared as described for 5a by using 1-bromo-2-phenylethane (3.28 mL, 24.02 mmol) and thioglycolic acid (2.00 mL, 28.79 mmol) in MeOH (10 mL), and a solution of NaOH (2.19 g, 54.78 mmol) in MeOH (40 mL). Yield 5b: 87% (4.10 g, 20.91 mmol) as white solid. ^1^H NMR (360 MHz, DMSO-d_6_): δ 12.57 (s, 1H, –COO*H*), 7.31–7.18 (m, 5H, H-2^II^, H-3^II^, H-4^II^), 3.26 (s, 2H, H-2), 2.83 (s, 4H, H-1^I^, H-2^I^). The spectroscopic data was consistent with the literature^33^.

#### ((3-Phenylpropyl)sulfanyl)acetic acid, 5c

Compound 5c was prepared as described for 5a by using 1-bromo-3-phenylpropane (5.45 mL, 35.86 mmol), thioglycolic acid (3.00 mL, 43.18 mmol) in methanol (20 mL), and a solution of NaOH (3.31 g, 82.76) in MeOH (60 mL). Yield 5c: 95% (7.18 g, 34.13 mmol) as a white solid. ^1^H NMR (360 MHz, DMSO-d_6_): δ 12.55 (s, 1H, –COO*H*), 7.30–7.16 (m, 5H, H-2^II^, H-3^II^, H-4^II^), 3.23 (s, 2H, H-2), 2.65 (t, *J*_1_I_,2_I = 7.4 Hz, 2H, H-1^I^), 2.58 (t, *J*_3_I_,2_I = 7.3 Hz, 2H, H-3^I^), 1.82 (m, *J*_2_I_,1_I = *J*_2_I_,3_I = 7.5 Hz, 2H, H-2^I^). The spectroscopic data was consistent with the literature^33^.

#### 1-(2-Nitro.4-(trifluoromethyl)phenyl)-1*H*-1,2,4-triazole, 2a

To a solution of 1,2,4-triazole (2.04 g, 29.48 mmol) in DMF (3.6 mL) was added 4-chloro-3-nitrobenzotrifluoride (4.0 mL, 27.22 mmol) and was heated to 100–105 °C. Once reached that temperature, K_2_CO_3_ (6.40 g, 48.31 mmol) was added slowly and the mixture was stirred at 115 °C during 30 min. Then, it was cooled to room temperature and 20 mL of water was added. The reaction crude and was extracted with AcOEt (4 × 20 mL) and the combined extracts were cleaned with water (3 × 15 mL) and brine (15 mL). Finally, the organics were dried with anhydrous Na_2_SO_4_ filtered and concentred under vacuum. An orange solid was obtained identified as 1-(2-nitro-4-(trifluoromethyl)phenyl)-1*H*-1,2,4-triazole, 2a (3.21 g, 12.4 mmol, 94% yield). Mp = 67–69 °C (from CH_2_Cl_2_). ^1^H NMR (360 MHz, CD_2_Cl_2_): δ 8.51 (s, 1H, H-5), 8.28 (s, 1H, H-3^I^), 8.12 (s, 1H, H-3), 8.05 (d, *J*_5_I_,6_I = 7.2 Hz, 1H, H-5^I^), 7.81 (d, *J*_6_I_,5_I = 7.2 Hz, 1H, H-6^I^). ^19^F NMR (250 MHz, CDCl_3_): δ 63.39 (s, –CF_3_). ^13^C NMR (90 MHz, CD_2_Cl_2_): δ 153.9 (C_3_), 144.4 (C_2_I), 144.2 (C_5_), 133.1 (C_1_I), 132.4 (q, *J*_4_I_,F_ = 34.2 Hz, C_4_I), 130.9 (q, *J*_5_I_,F_ = 4.5 Hz, C_5_I), 128.0 (C_6_I), 123.5 (q, *J*_3_I_,F_ = 3.6 Hz, C_3_I), 122.7 (q, *J*_1_II_,F_ = 271.8 Hz, –*C*F_3_). IR (ATR): 2999, 1543, 1363, 1322, 1184, 1135, 1036, 979, 841 cm^−1^. HRMS (ESI +): calcd. for [C_9_H_5_F_3_N_4_O_2_ + H]^+^: 259.0443, found [M + H]^+^ 259.0442.

#### 1-(4-Methyl-2-nitrophenyl)-1*H*-1,2,4-triazole, 2b

To a solution of 1,2,4-triazole (137 mg, 1.98 mmol) in DMF (0.25 mL) was added 4-chloro-3-nitrotoluene (0.24 mL, 1.80 mmol) and was heated to 105 °C. Once reached that temperature, K_2_CO_3_ (438 mg, 3.08 mmol) was added slowly and the mixture was heated to 115 °C and stirred for 6 h. Then, another portion of 1,2,4-triazole (130 mg, 1.88 mmol) and DMF (0.25 mL) was added and the mixture was stirred overnight. The crude was cooled to room temperature and poured into a beaker with water/ice (10 mL). The resulting suspension was extracted with EtOAc (3 × 15 mL) and cleaned with and brine (15 mL). Finally, the organic layers were dried with anhydrous Na_2_SO_4_, filtered and concentred under vacuum. The resulting red oil was purified by column chromatography (EtOAc/hexane, 1:1) to obtain a yellow solid identified as 1-(4-methyl-2-nitrophenyl)-1H-1,2,4-triazole, 2b (301 mg, 1.47 mmol, 82% yield). Mp = 67–69 °C (from CH_2_Cl_2_). ^1^H NMR (400 MHz, CDCl_3_): δ 8.36 (s, 1H, H-5), 8.09 (s, 1H, H-3), 7.83 (m, *J*_3_I_,5_I = 1.2 Hz, 1H, H-3^I^), 7.54 (dm, *J*_5_I_,6_I = 8.1 Hz, *J*_5_I_,3_I = 1.2 Hz, 1H, H-5^I^), 7.44 (d, *J*_6_I_,5_I = 8.1 Hz, 1H, H-6^I^). ^13^C NMR (100 MHz, CDCl_3_): δ 153.1 (C_3_), 144.5 (C_2_I), 144.0 (C_5_), 141.7 (C_4_I), 134.3 (C_5_I), 127.9 (C_1_I), 127.5 (C_6_I), 125.9 (C_6_I), 21.2 (–*C*H_3_). IR (ATR): 3113, 1533, 1355, 1278, 1214, 1142, 1042, 985, 837 cm^−1^. HRMS (ESI +): calcd. for [C_9_H_8_N_4_O_2_ + H]^+^: 205.0726, found [M + H]^+^ 205.0724.

#### 2-(1*H*-1,2,4-triazol-1-yl)-5-(trifluoromethyl)aniline, 3a

Compound 2a (9.98 g, 38.65 mmol) and Na_2_S (3.02 g, 38.6 mmol) were dissolved with a 1:1 degassed mixture of dioxane and water (140 mL) under N_2_ atmosphere. The reaction mixture was heated to 75–80 °C and stirred over 2 h. Another portion of Na_2_S (1.50 g, 19.25 mmol) was added and the mixture was stirred 1 h. Then, the reaction was quenched with a saturated aqueous solution of NaHCO_3_ (15 mL) and the crude product was extracted with AcOEt (3 × 30 mL) and the combined organic layers were cleaned with water (3 × 20 mL) and brine (20 mL). Then, it was dried over anhydrous Na_2_SO_4_ and the solvent was removed under vacuum. The resulting orange oil was purified by column chromatography (AcOEt/hexane, 1:1 → 1:2) to furnish a yellow solid identified as 2-(1*H*-1,2,4-triazole-1-yl)-5-(trifluoromethyl)aniline, 3a (4.48 g, 19.65 mmol, 51% yield). Mp = 122–123 °C (from CH_2_Cl_2_). ^1^H NMR (360 MHz, CD_2_Cl_2_): δ 8.44 (s, 1H, H-5^I^), 8.15 (s, 1H, H-3^I^), 7.34 (d, *J*_3,4_ = 8.3 Hz, 1H, H-3), 7.13 (s, 1H, H-6), 7.04 (d, *J*_4,3_ = 8.3 Hz, 1H, H-4), 4.97 (s, 2H, –N*H*_2_). ^19^F NMR (250 MHz, CDCl_3_): δ 63.53 (s, –CF_3_). ^13^C NMR (90 MHz, CD_2_Cl_2_): δ 153.1 (C_3_I), 144.1 (C_5_^I^), 142.0 (C_1_), 132.0 (q, *J*_5,F_ = 33.5 Hz, C_5_), 125.3 (C_2_), 125.0 (C_3_), 124.3 (q, *J*_1_II_,F_ = 270.5 Hz, –*C*F_3_), 114.8 (q, *J*_4,F_ = 4.1 Hz, C_4_), 114.6 (q, *J*_6,F_ = 3.5 Hz, C_6_). IR (ATR): 3432, 3320, 1635, 1456, 1344, 1271, 1149, 1102, 958 cm^−1^. HRMS (ESI +): calcd. for [C_9_H_7_F_3_N_4_ + H]^+^: 229.0701, found [M + H]^+^ 229.0701.

#### 5-methyl-2-(1*H*-1,2,4-triazol-1-yl)aniline, 3b

Compound 2b (5.00 g, 24.49 mmol) and Na_2_S (2.87 g, 36.77 mmol) were dissolved with a 1:1 degassed mixture of dioxane and water (100 mL) under N_2_ atmosphere. The reaction mixture was heated to 75 °C and stirred over 1 h. Then, a second portion of Na_2_S (956 mg, 12.25 mmols) was added and the mixture was stirred 1 h. The reaction was quenched with a saturated aqueous solution of NaHCO_3_ (15 mL) and the crude product was extracted with AcOEt (3 × 100 mL) and the combined organic layers were cleaned with water (2 × 150 mL) and brine (250 mL). Then, it was dried over anhydrous Na_2_SO_4_ and the solvent was removed under vacuum. The resulting yellow solid was purified by column chromatography (AcOEt/hexane, 2:1) to furnish a yellow solid identified as 5-methyl-2-(1*H*-1,2,4-triazol-1-yl)aniline 3b (2.91 g, 16.70 mmol, 60% yield). Mp = 67–69 °C (from CH_2_Cl_2_). ^1^H NMR (400 MHz, CDCl_3_): δ 8.31 (s, 1H, H-5^I^), 8.12 (s, 1H, H-3^I^), 7.05 (d, *J*_3,4_ = 8.0 Hz, 1H, H-3), 6.66 (s, 1H, H-6), 6.66 (d, *J*_4,3_ = 8.0 Hz, 1H, H-4), 4.41 (s, 2H, –N*H*_2_), 2.30 (s, 3H, –CH_3_). ^13^C NMR (100 MHz, CDCl_3_): δ 152.4 (C_3_I), 143.4 (C_5_^I^), 140.9 (C_1_), 140.4 (C_5_), 124.3 (C_3_), 120.7 (C_2_), 119.3 (C_4_), 117.9 (C_6_), 21.3 (–*C*H_3_). IR (ATR): 3422, 3336, 3118, 1631, 1518, 1276, 1137, 956 cm^−1^. HRMS (ESI +): calcd. for [C_9_H_10_N_4_ + H]^+^: 175.0984, found [M + H]^+^ 175.0980.

#### *N*-(2-(1*H*-1,2,4-triazol-1-yl)-5-(trifluoromethyl)phenyl)-2-(benzylthio)acetamide, B1

To a solution of compound 5a (807 mg, 4.43 mmol), and DMF (4 drops) in dry CH_2_Cl_2_ (8 mL) under N_2_ atmosphere, SOCl_2_ (0.95 mL, 13.09 mmol) was added dropwise, and the mixture was heated to reflux conditions during 4 h. After that, the solvent was removed under reduced pressure and the crude was redissolved in dry CH_2_Cl_2_ (8 mL), and the corresponding solution concentrated again under reduced pressure. This operation was repeated 3 times in order to remove the traces of SOCl_2_ and the obtained acyl chloride was dissolved in anhydrous ACN (10 mL) under N_2_ atmosphere conditions. In another round bottom flask, DIPEA (0.77 mL, 4.42 mmol) was added to a solution of compound 3a (501 mg, 2.20 mmol) in dry ACN (10 mL) under N_2_ atmosphere. This mixture was heated to the reflux temperature, and the acyl chloride solution was slowly added in small portions (1.5 mL) every 15 min. Next, the mixture was let stirred overnight at room temperature. The reaction was quenched with water (30 mL) and diluted with CH_2_Cl_2_ (30 mL). The organic layer was separated and cleaned with water (2 × 30 mL) and brine (30 mL), dried with anhydrous Na_2_SO_4_ and removed under vacuum. The brown oil obtained was purified by column chromatography (CH_2_Cl_2_/Et_2_O, 2;1) to furnish a pale brown solid identified as *N*-(2-(1*H*-1,2,4-triazol-1-yl)-5-(trifluoromethyl)phenyl)-2-(benzylthio)acetamide, B1 (713 mg, 1.82 mmol, 83% yield). Mp = 104–107 °C (from CH_2_Cl_2_). ^1^H NMR (360 MHz, CD_2_Cl_2_): δ 10.51 (s, 1H, –*H*NCO–), 8.82 (s, 1H, H-6^III^), 8.53 (s, 1H, H-5^IV^), 8.24 (s, 1H, H-3^IV^), 7.52 (m, *J*_3_III_,4_III = 8.3 Hz, 1H, H-3^III^), 7.50 (m, *J*_4_III_,3_III = 8.3 Hz, 1H, H-4^III^), 7.28–7.16 (m, 5H, 2xH-2^II^, 2xH-3^II^, H-4^II^), 3.72 (s, 2H, H-1^I^), 3.27 (s, 2H, H-2). ^19^F NMR (250 MHz, CDCl_3_): δ 63.30 (s, –CF_3_). ^13^C NMR (90.5 MHz, CD_2_Cl_2_): δ 168.1 (C_1_), 153.6 (C_3_IV), 144.3 (C_5_IV), 137.2 (C_1_II), 132.6 (C_1_II), 131.5 (q, *J*_5_III,_F_ = 32.5 Hz, C_5_III), 129.4 (C_2_II), 128.9 (C_3_II), 128.7 (C_2_III), 127.7 (C_4_II), 123.9 (q, *J*_1_IV_,F_ = 270.9 Hz, C_1_IV), 123.9 (C_3_III), 121.4 (q, *J*_4_III_,F_ = 3.9 Hz, C_4_III), 120.2 (q, *J*_6_III_,F_ = 4.1 Hz, C_6_III), 37.4 (C_1_I), 37.0 (C_2_)_._ IR (ATR): 3286, 1687, 1531, 1476, 1435, 1330, 1132 cm^−1^. HRMS (ESI +): calcd. for [C_18_H_15_F_3_N_4_OS + H]^+^: 393.0997, found [M + H]^+^ 393.0991.

#### *N*-(2-(1*H*-1,2,4-triazol-1-yl)-5-(trifluoromethyl)phenyl)-2-(phenethylthio)acetamide, B2

Compound B2 was prepared as described for B1 by using compound 5b (739 mg, 3.94 mmol), SOCl_2_ (0.93 mL, 12.86 mmol) and DMF (5 drops) in dry CH_2_Cl_2_ (7 mL). The resulting acyl chloride was dissolved in dry ACN (10 mL) and was added to a solution of compound 3a (224 mg, 0.98 mmol), DIPEA (0.35 mL, 2.04 mmol) in dry ACN (5 mL). Yield B2: 60% (238 mg, 0.59 mmol) as pale-yellow solid. Mp = 70–72 °C (from CH_2_Cl_2_). ^1^H NMR (400 MHz, CD_2_Cl_2_): δ 10.60 (s, 1H, –*H*NCO–), 8.89 (s, 1H, H-6^III^), 8.51 (s, 1H, H-5^IV^), 8.22 (s, 1H, H-3^IV^), 7.55 (m, *J*_3_III_,4_III = 8.4 Hz, 1H, H-3^III^), 7.51 (m, *J*_4_III_,3_III = 8.5 Hz, 1H, H-4^III^), 7.30–7.17 (m, 5H, 2xH-2^II^, 2xH-3^II^, H-4^II^), 3.37 (s, 2H, H-2), 2.86 (s, 2H, H-2^I^), 2.77 (s, 2H, H-1^I^). ^19^F NMR (250 MHz, CDCl_3_): δ 63.29 (s, –CF_3_). ^13^C NMR (100.6 MHz, CD_2_Cl_2_): δ 168.5 (C_1_), 153.8 (C_3_IV), 144.4 (C_5_IV), 140.4 (C_1_II), 132.9 (C_1_III), 131.8 (q, *J*_5_III,_F_ = 33.2 Hz, C_5_III), 129.0 (5C, C_2_III, 2xC_2_II, 2xC_3_II), 127.0 (C_4_II), 124.3 (C_3_III), 124.1 (q, *J*_1_IV_,F_ = 273.0 Hz, C_1_IV), 121.6 (q, *J*_4_III_,F_ = 4.1 Hz, C_4_III), 120.4 (q, *J*_6_III_,F_ = 4.1 Hz, C_6_III), 37.9 (C_2_), 36.0 (C_2_I), 34.8 (C_1_I). IR (ATR): 3275, 1687, 1536, 1479, 1434, 1333, 1124 cm^−1^. HRMS (ESI +): calcd. for [C_19_H_17_F_3_N_4_OS + H]^+^: 407.1153, found [M + H]^+^ 407.1151.

#### *N*-(2-(1*H*-1,2,4-triazol-1-yl)-5-(trifluoromethyl)phenyl)-2-((3-phenylpropyl)thio)acetamide, B3

Compound B3 was prepared as described for B1 by using compound 5c (832 mg, 3.95 mmol), SOCl_2_ (0.75 mL, 10.38 mmol) and DMF (5 drops) in dry CH_2_Cl_2_ (8 mL). The resulting acyl chloride was dissolved in dry ACN (10 mL) and was added to a solution of compound 3a (448 mg, 1.96 mmol), DIPEA (1 mL, 5.74 mmol) in dry ACN (10 mL). Yield B3: 56% (463 mg, 1.10 mmol) as a pale-yellow solid. Mp = 78–80 °C (from CH_2_Cl_2_). ^1^H NMR (400 MHz, CD_2_Cl_2_): δ 10.60 (s, 1H, –*H*NCO–), 8.87 (s, 1H, H-6^III^), 8.48 (s, 1H, H-5^IV^), 8.24 (s, 1H, H-3^IV^), 7.55 (m, *J*_3_III_,4_III = 8.4 Hz, 1H, H-3^III^), 7.52 (m, *J*_4_III_,3_III = 8.5 Hz, *J*_4_III_,6_III = 1.8 Hz, 1H, H-4^III^), 7.26–7.13 (m, 5H, 2xH-2^II^, 2xH-3^II^, H-4^II^), 3.37 (s, 2H, H-2), 2.66 (m, *J*_3_I_,2_I = 7.4 Hz, 2H, H-3^I^), 2.51 (m, *J*_1_I_,2_I = 7.6 Hz, 2H, H-1^I^), 1.86 (m, *J*_2_I_,1_I_/3_I = 7.6 Hz, 2H, H-2^I^). ^19^F NMR (250 MHz, CDCl_3_): δ 63.27 (s, ^−^CF_3_). ^13^C NMR (100.6 MHz, CD_2_Cl_2_): δ 168.7 (C_1_), 153.8 (C_3_IV), 144.4 (C_5_IV), 141.8 (C_1_II), 132.9 (C_2_III), 131.8 (q, *J*_5_III,_F_ = 32.9 Hz, C_5_III), 129.0 (C_1_III), 128.9 (5C, 2xC_2_II, 2xC_3_II, C_4_II), 124.3 (C_3_III), 124.1 (q, *J*_1_IV_,F_ = 272.0 Hz, –*C*F_3_), 121.6 (q, *J*_4_III_,F_ = 4.2 Hz, C_4_III), 120.4 (q, *J*_6_III_,F_ = 4.2 Hz, C_6_III), 37.8 (C_2_), 35.0 (C_3_I), 32.8 (C_1_I), 31.1 (C_2_I). IR (ATR): 3186, 1690, 1533, 1479, 1426, 1330, 1170, 1127 cm^−1^. HRMS (ESI +): calcd. for [C_20_H_19_F_3_N_4_OS + H]^+^: 421,1310, found [M + H]^+^ 421,1308.

#### 2-(benzylthio)-*N*-(5-methyl-2-(1*H*-1,2,4-triazol-1-yl)phenyl)acetamide, B4

To a solution of compound 5a (514 mg, 2.82 mmol) and HATU (1.049 g, 2.76 mmol) in anhydrous THF (16 mL) and anhydrous DMF (2 mL), DIPEA (1.5 mL, 7.6 mmol) was added under N_2_ atmosphere and the mixture was stirred for 30 min. Then, another solution of amine 3b (307 mg, 1.76 mmol) in anhydrous THF (5 mL) under N_2_ atmosphere was prepared and added to the previous solution. The reacting mixture was stirred overnight, quenched with water (20 mL) and extracted with CH_2_Cl_2_ (3 × 25 mL). The combined organic extracts were cleaned with brine (75 mL), dried with Na_2_SO_4_, filtered and concentrated under reduced pressure. The obtained brown oil was purified by column chromatography (EtOAc/hexane, 1:1) to furnish a white solid identified as 2-(benzylthio)-*N*-(5-methyl-2-(1*H*-1,2,4-triazol-1-yl)phenyl)acetamide, B4 (401 mg, 1.19 mmol, 60% yield). Mp = 104–106 °C (from CH_2_Cl_2_). ^1^H NMR (400 MHz, acetone-d_6_): δ 9.97 (s, 1H, –*H*NCO–), 8.79 (s, 1H, H-5^IV^), 8.22 (s, 1H, H-6^III^), 8.22 (s, 1H, H-3^IV^), 7.47 (m, *J*_3_III_,4_III = 8.1 Hz, 1H, H-3^III^), 7.29–7.19 (m, 5H, 2xH-2^II^, 2xH-3^II^, H-4^II^), 7.11 (m, *J*_4_III_,3_III = 8.1 Hz, 1H, H-4^III^), 3.75 (s, 2H, H-1^I^), 3.26 (s, 2H, H-2), 2.41 (s, 3H, H-1^ V^). ^13^C NMR (100.6 MHz, acetone-d_6_): δ 168.0 (C_1_), 153.5 (C_3_IV), 145.3 (C_5_IV), 140.3 (C_5_III), 138.4 (C_1_II), 132.7 (C_1I_II), 129.9 (C_2_II), 129.3 (C_3_II), 127.9 (C_4_II), 125.9 (C_4_III), 125.0 (C_3_III), 124.1 (C_6_III), 37.0 (C_1_I), 36.5 (C_2_), 36.5 (–*C*H_3_). IR (ATR): 3273, 1673, 1592, 1538, 1498, 1374, 1141 cm^−1^. HRMS (ESI +): calcd. for [C_18_H_18_N_4_OS + Na]^+^: 361.1099, found [M + Na]^+^ 361.1097.

#### *N*-(5-methyl-2-(1*H*-1,2,4-triazol-1-yl)phenyl)-2-(phenethylthio)acetamide, B5

Compound B5 was prepared as described for B4 by using a solution of compound 5b (518 mg, 2.64 mmol), HATU (1.00 g, 2.63 mmol) and DIPEA (1.2 mL, 6.9 mmol) in anhydrous THF (17 mL) and anhydrous DMF (2 mL). The activated acid solution was added to a solution of amine 3b (306 mg, 1.76 mmol) in dry THF (8 mL). Yield B5: 83% (514 mg, 1.46 mmol) as a white solid. Mp = 109–110 °C (from CH_2_Cl_2_). ^1^H NMR (360 MHz, CD_2_Cl_2_): δ 10.07 (s, 1H, –*H*NCO–), 8.39 (s, 1H, H-5^IV^), 8.26 (s, 1H, H-6^III^), 8.15 (s, 1H, H-3^IV^), 7.30–7.16 (m, 6H, H-3^III^, 2xH-2^II^, 2xH-3^II^, H-4^II^), 7.07 (m, *J*_4_III_,3_III = 8.1 Hz, 1H, H-4^III^), 3.31 (s, 2H, H-2), 2.81 (s, 2H, H-2^I^), 2.71 (s, 2H, H-1^I^), 2.43 (s, 1H, –C*H*_3_). ^13^C NMR (90.5 MHz, CD_2_Cl_2_): δ 168.0 (C_1_), 153.3 (C_3_IV), 144.3 (C_5_IV), 140.8 (C_5_II), 140.5 (C_1_II), 132.1 (C_1_III), 129.0 (4C, 2xC_2_II, 2xC_3_II), 126.9 (C_4_II), 125.8 (C_3_III), 124.8 (C_2_III), 124.0 (C_4_III), 123.8 (C_6_III), 37.8 (C_2_), 36.0 (C_2_I), 34.8 (C_1_I), 21.8 (–*C*H_3_). IR (ATR): 3277, 1683, 1588, 1529, 1482, 1280 cm^−1^. HRMS (ESI +): calcd. for [C_19_H_20_N_4_OS + H]^+^: 353.1436 m/z [M + H]^+^; found 353.1433.

#### *N*-(5-methyl-2-(1*H*-1,2,4-triazol-1-yl)phenyl)-2-(phenypropylthio)acetamide, B6

Compound B6 was prepared as described for B4 by using a solution of compound 5c (197 mg, 0.94 mmol), HATU (360 m g, 0.94 mmol) and DIPEA (0.44 mL, 2.50 mmol) in anhydrous THF (5 mL) and anhydrous DMF (1 mL). The activated acid solution was added to a solution of amine 3b (109 mg, 0.63 mmol) in dry THF (5 mL). Yield B6: 50% (120 mg, 0.31 mmol) as a white solid. Mp = 78–80 °C (from CH_2_Cl_2_). ^1^H NMR (400 MHz, CD_2_Cl_2_): δ 10.06 (s, 1H, –*H*NCO–), 8.35 (s, 1H, H-5^IV^), 8.24 (s, 1H, H-6^III^), 8.16 (s, 1H, H-3^IV^), 7.27–7.14 (m, 6H, H-3^III^, 2xH-2^II^, 2xH-3^II^, H-4^II^), 7.07 (m, *J*_4_III_,3_III = 8.2 Hz, *J*_4_III_,6_III = 1.2 Hz, 1H, H-4^III^), 3.30 (s, 2H, H-2), 2.65 (m, *J*_3_I_,2_I = 7.5 Hz, 2H, H-3^I^), 2.46 (m, *J*_1_I_,2_I = 7.6 Hz, 2H, H-1^I^), 2.44 (s, 3H, H-1^ V^), 1.84 (m, *J*_2_I_,1_I_/3_I = 7.5 Hz, 2H, H-2^I^). ^13^C NMR (100.6 MHz, CD_2_Cl_2_): δ 168.1 (C_1_), 153.4 (C_3_IV), 144.3 (C_5_IV), 141.9 (C_1_II), 140.8 (C_5_III), 132.2 (C_1_III), 129.0 (C_2_II), 128.9 (C_2_II), 126.5 (C_4_II), 125.8 (C_4_III), 124.9 (C_2_III), 124.1 (C_3_III), 123.9 (C_6_III), 37.8 (C_2_), 35.1 (C_3_I), 32.8 (C_1_I), 31.1 (C_2_I), 21.8 (–*C*H_3_). IR (ATR): 2946, 1688, 1593, 1542, 1509, 1414, 1283 cm^−1^. HRMS (ESI +): calcd. for [C_20_H_22_N_4_OS + H]^+^: 367.1593, found [M + H]^+^ 367.1591.

### cAMP levels in culture medium

HEK293T cells (ATCC-CRL-11268, American-type culture collection, United States) were grown onto 60-mm diameter culture dishes for 24 h in Dulbecco’s Modified Eagle’s medium (DMEM) supplemented with 10% of fetal bovine serum (FBS), in a humidified atmosphere at 95% air and 5% CO_2_. Cells were transfected using Lipofectamin 2000 (Invitrogen Life Technologies) following provider’s instructions (1 µg DNA/3 µl Lipofectamin 2000). 24 h later, cells were transferred to 24-well plates at 150,000 cells/well and incubated for 24 h. Cells were incubated for 30 min at 37 °C with 10 µM forskolin in presence or absence of allatostatin and/or antagonist (B5 or B6) in 0.5 ml DMEM. The cells were then washed with phosphate-buffered saline and lysed. The intracellular levels of cyclic adenosine monophosphate (cAMP) were determined with a DetectX® Cyclic AMP (cAMP) Direct Immunoassay kit (K019-H1; Arbor Assays, Ann Arbor, MI, USA) according to the manufacturer instructions.

### Behavioral assays

Honey bees (*Apis mellifera*, Buckfast) were collected daily from colonies at the experimental apiary {~ 30 hives) located on the campus of Toulouse University. They were taken from different colonies from one day to another, in order to take into account inter-colony variability (due to e.g. genetically different queens, colony size or health status). Experiments were conducted between May 2018 and July 2019. After collection, they were cold-anesthetized and mounted in restraining holders, then fed with 5 μL sucrose (50% in water) before proceeding. During resting periods between manipulations, they were kept in the dark at room temperature. All chemicals (except the treatment solutions) were purchased from Sigma (Lyon, France).

*Assessment of learning performance.* Bees were injected with A8 diluted in phosphate buffer saline (PBS, Sigma-Aldrich) or PBS alone for controls (200 nL in the head capsule), 1 h after feeding. One hour later, they were exposed for 15 min to isopentyl acetate (IPA, 24% in paraffin oil), and underwent a 3-trial olfactory conditioning 45 min later. Briefly, on each trial they were trained to associate an odorant (1-hexanol or 1-nonanol, presented for 4 s) with a sucrose reward (delivered for 3 s). Extension of the proboscis (mouthparts) during the odor presentation and before the reward delivery was counted as a conditioned response. Details of the protocol can be found elsewhere^6^. Bees not responding to sucrose alone were discarded.

*Assessment of appetitive response.* One hour after feeding, bees were injected with one of the treatment solutions (200 nL in the head capsule) then exposed to IPA (as above) 1 h later. The appetitive response was assessed 45 min after exposure (i.e. 3 h following feeding). For this, the proboscis extension reflex (PER) was tested in response to applying increasing sucrose concentrations (0.1, 0.3, 1, 3, 10, 30%) on the antennae, and the presence or absence of a full extension of the proboscis was noted for each concentration. Since sucrose was diluted in water, bees were allowed to drink water ad libitum before the test, and antennal applications of water were interspersed with sucrose presentations to ensure that the responses were specifically elicited by sucrose. All bees responding to water were discarded.

### Statistical analysis

Sample sizes are provided in the figure legends. Learning performance was measured, for each treatment group, as the percentage of bees displaying a conditioned response at the corresponding trial. The effect of treatment on learning performance was assessed using a one-factor ANOVA on the proportions of conditioned responses by the end of conditioning (3rd trial). Appetitive responses were analyzed in two ways. For each treatment, an individual sucrose responsiveness score (SRS, ranging between 0 and 6) was calculated as the sum of PER displayed over the 6 sucrose presentations. For each tested molecule, effects of molarity were assessed by comparing percentages of responses to increasing sucrose concentrations with a repeated-measure ANOVA, as well as average SRS values with a one-factor ANOVA. We applied an arcsin transformationof the data before applying ANOVA, and multiple comparisons were applied using a Bonferroni correction.

## Results

### Screening of ligands of the ASTA receptor

**Figure 1:**
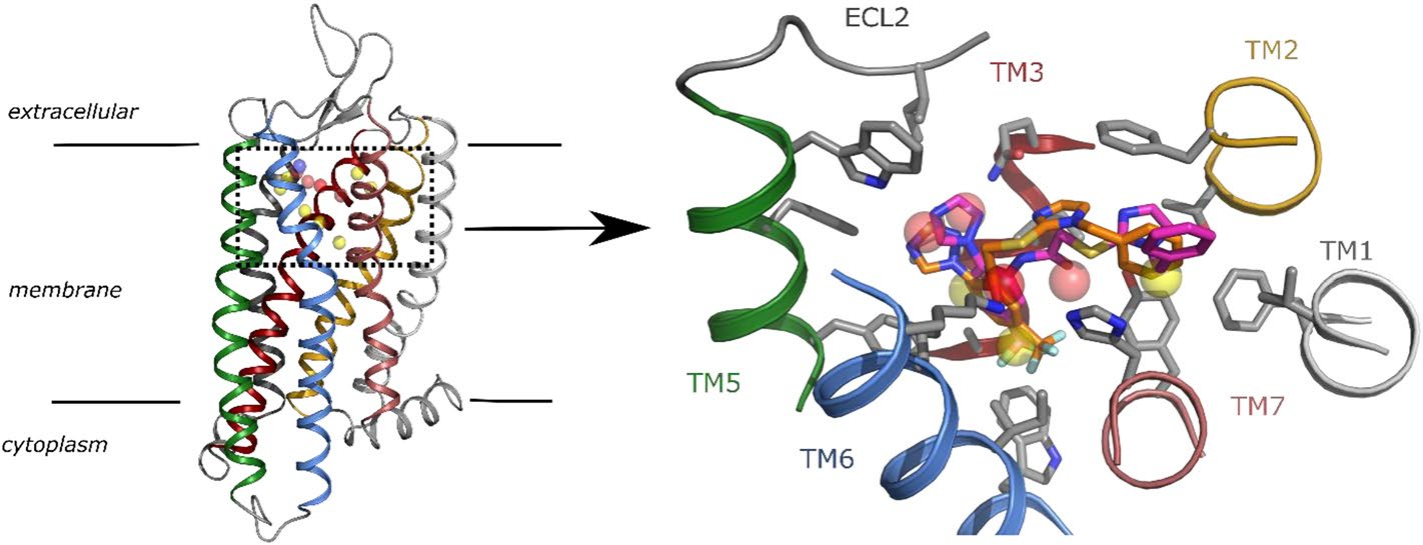
ASTA Receptor model and pharmacophore. (Left) Cartoon representation of the homology model of the ASTA-R with transmembrane helices displayed in different colors. The dotted rectangle indicates the localization of the typical orthosteric binding pocket in class A GPCRs (zoomed in the right panel). Spheres represent the 13 pharmacophoric features defined for the virtual screening (yellow: hydrophobic, red: hydrogen bond acceptor, purple: negative charge). (Right) Zoom of the orthosteric pocket of ASTA displaying the best two docking poses of compound A8 (colored by atom type with carbons in orange or magenta). Spheres represent the 7 pharmacophoric features matched by compound A8 in the virtual screening. Helices are shown as cartoon and residue side-chains around A8 as sticks (colored by atom type with carbons in grey). ECL: extracellular loop; TM: transmembrane helix.

We aimed to perform a virtual screening to find non-peptidic ligands for the honey bee allatostatin A receptor (ASTA-R). With this purpose, we created a homology model of the receptor based on the structure of the human δ-opioid receptor^30^, since no allatostatin receptor structures are yet available (see Methods). We used electrostatic probes on the area that corresponds to the typical orthosteric binding pocket of class A GPCRs to define possible requirements (a pharmacophore model) of putative ASTA-R ligands (Fig. 1).

We screened a subset of the ZINC database^32^ as described in the Methods section. The fact that we use a model based on an inactive receptor should favor identifying antagonists. Yet, because it is not known if any of the pharmacophoric features used is associated with agonism or antagonism, hits could be either agonists or antagonists. After visual inspection of the best 1000 ranked compounds, we selected 24 molecules for experimental testing (‘A compounds’, Sup. Table 1).

**Figure 2:**
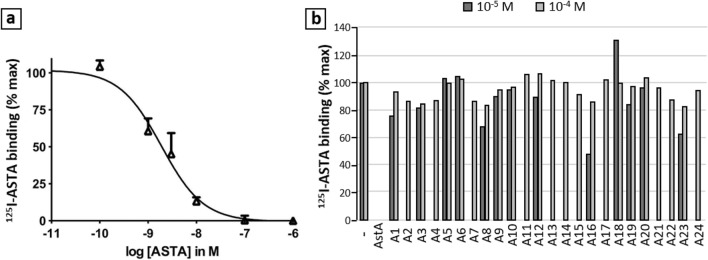
In vitro competitive binding assays for the A-series molecules. (**a**) Competition curve for the native ligand ASTA on HEK cells. (**b**) Results (% max) obtained with the 24 tested A molecules (positive control: ASTA 10^-6^ M). Only A8, A16 and A23 showed dose-dependent binding (3 replicates).

All 24 compounds were tested for their capacity to compete with ^125^I-ASTA on HEK 293 cells transfected with the ASTA receptor (Fig. 2). While ASTA had an IC_50_ in the nanomolar range (Fig. 2a), consistently with previous data^6^, only 3 of the tested compounds were able to compete in a dose-dependent manner, with low micromolar affinities: A8, A16 and A23 (Fig. 2b). These active molecules were used to optimize the ligand binding modes in the receptor model, and were selected for testing in vivo.

### Modulation of behavioral manifestations of stress

**Figure 3:**
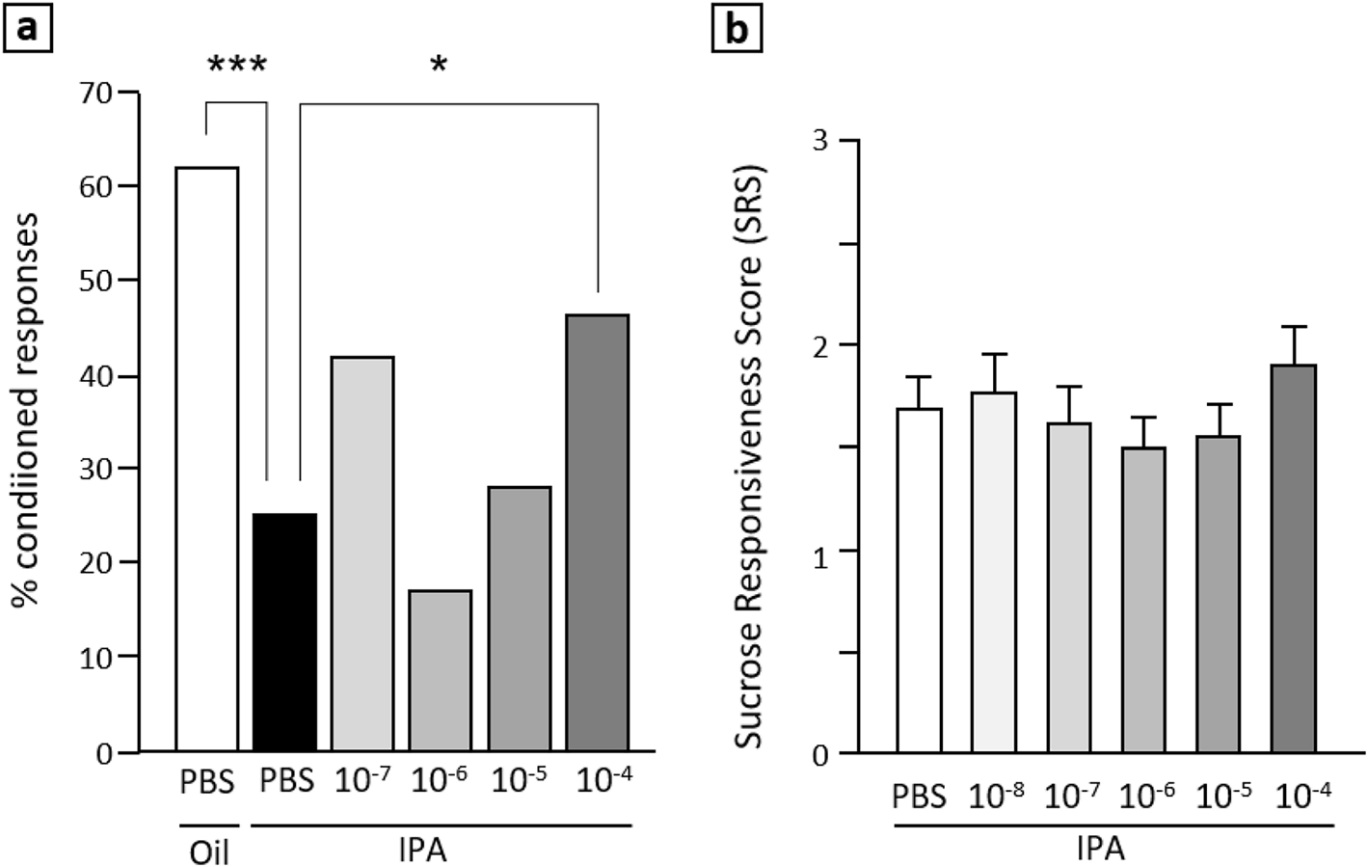
Effects of A8 on stressed bees. (**a**) Learning: proportions of individuals showing a conditioned response to the odorant paired with sucrose, at the end of the conditioning session (third trial). Bees were injected either with PBS or A8, then exposed to IPA or paraffin oil only (negative controls). While exposure to IPA significantly reduced learning performance in PBS-injected bees, this effect was restored by an injection of 10^–4^ M A8. *: *p* < 0.025; ***p < 0.0005 (PBS: n = 100; A8: n = 47–99/group). (**b**) Sucrose responsiveness: mean sucrose responsiveness score values in bees injected with PBS or A8 before exposure to IPA. Even at the highest dose, A8 could not significantly increase SRS as compared to PBS controls (PBS: n = 78; A8: n = 44–47/group) (2 replicates).

We ran experiments to test the capacity of A8, A16 and A23 to act as a possible antagonists of ASTA-R in vivo, and thus to limit behavioral manifestations of the stress response. For this, we used the exposure to IPA (the main component of alarm pheromone) as the stress condition. Our previous work showed that exposure to IPA reduces both the learning performance and the probability of appetitive responses. We thus tested whether treating bees with either compound prior to exposure could reduce the impact of this stressor on behavior. First, bees were exposed to IPA following injections of the tested molecule, then submitted to a classical conditioning assay where they had to learn to respond to an odorant which was repeatedly delivered in association with a food reward. Two control groups injected with PBS: one was unstressed (exposed to oil only) and the other was exposed to IPA. We focused on A8 since neither A16 nor A23 appeared to have detectable effects in a preliminary experiment (Fig. S1). While, as expected, the stressed control PBS group displayed a marked decrease in learning as compared to its unstressed counterpart at the end of conditioning, the performance of A8-treated bees seemed to display a U-shape dose-dependent curve (*trial x dose* effect: F_3,104_ = 2.627, *p* = 0.0345) (Fig. 3).

The learning performances of bees receiving the highest and lowest A8 doses did not differ from those of negative controls (unstressed bees receiving PBS) (10^–7^ M: Z = 2.233, *p* = 0.0001; 10^–4^ M: Z = 2.025, *p* = 0.0048). However, only 10^–4^ M doses performed better than that of stressed bees (Z = 2.842, *p* = 0.0224) (Fig. 3a). Thus, high doses of A8 could restore the learning performance of stressed bees at a level comparable to that of unstressed bees. We then focused on A8 for further experiments and to optimize the ligand binding modes in the receptor model.

In the same experiment, we compared the proportions of bees that had to be discarded due to a lack of proboscis extension reflex in response to 50% sucrose (the unconditioned stimulus used in the learning task). Indeed, exposure to IPA markedly increased this proportion in PBS-injected bees (IPA/PBS: 38.5%; Oil/PBS: 1.9%; Z = 4.458, *p* < 0.001), consistently with previous works, indicating a reduced motivation for food ^24^. However, this effect was significantly attenuated following treatment with A8 at 10^–4^ M (IPA/10^–4^: 23.4%, Z = 2.249, *p* = 0.049). Thus, A8 improved the probability to respond to 50% sucrose, a coarse measure of appetitive motivation. In order to explore further the action of A8 on sucrose responsiveness, we used a standard protocol designed to provide a more sensitive assessment of appetitive motivation through the calculation of individual sucrose responsiveness scores (SRS). Following injection with A8 (from 10^–8^ to 10^–4^ M), then exposure to IPA, groups of bees were individually tested for their capacity to display an appetitive response upon antennal stimulation with increasing sucrose concentrations in a lower range (0.1–30%). As expected, bees were more prone to respond as the sucrose concentration increased (not shown), but this progression depended on the treatment (*dose x sucrose concentration* interaction: F_5,1335_ = 1.582, *p* = 0.035). Indeed, mean score values followed a U-shape curve, but none of the tested doses could significantly increase responsiveness (Fig. 3b), even though the higher score was that of bees treated with 10^−4^ M A8. Thus, the capacity for A8 to modulate appetitive motivation in stressed bees was observed only in response to high (50%) sucrose concentrations. Nevertheless, albeit at a high dose, A8 could partially restore learning performance and motivation for 50% sucrose in stressed bees.

### Chemical synthesis of a second generation of potential receptor ligands

**Figure 4:**
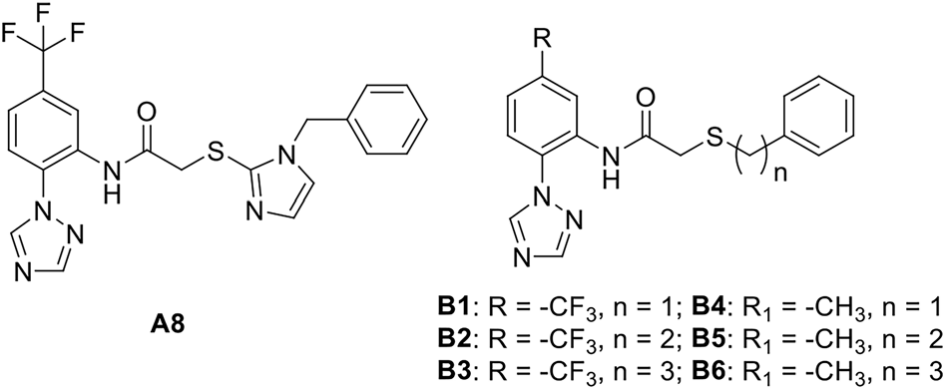
Structures of A8 and the related synthesized compounds B1-6.

**Figure 5:**
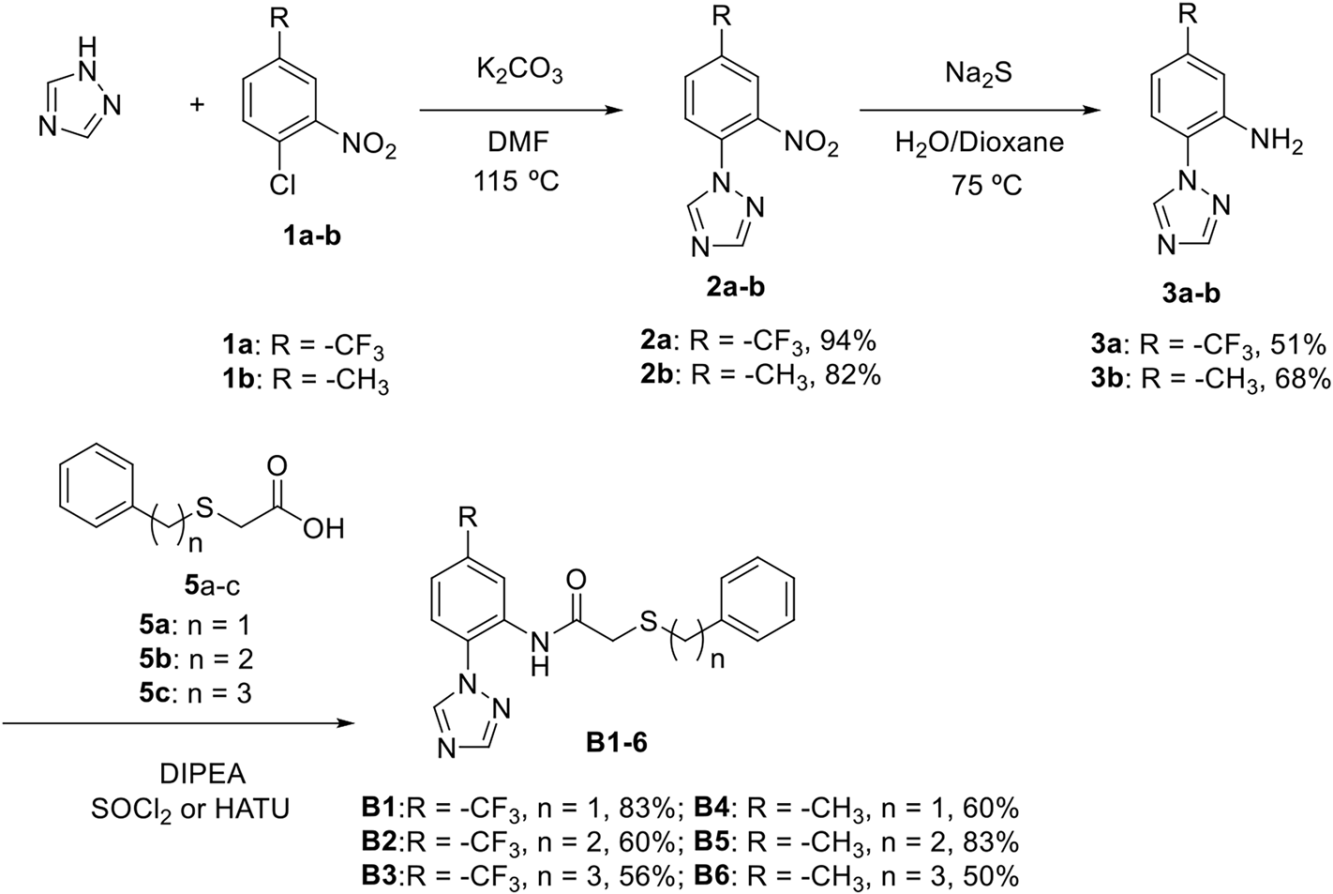
Synthesis of B1-6 target compounds.

We focused our synthetic efforts on target molecules structurally related to the A8 compound, which showed the highest impact on behavioral manifestations of stress. The main scaffold of the new candidate compounds (B series) retains the main features of A8 according to the pharmacophore model generated, while changing other parts of the molecule. Specifically, we planned to study the possible influence of two structural factors: the classical bioisostere substitution of the trifluoromethyl present in the phenyl ring of the 1-(phenyl)-1*H*-1,2,4-triazole bicyclic system of A8, by a methyl, in order to try to tune up the final activity of the molecule; and the length of the connecting chain (n = 1, 2 and 3) between the former bicyclic moiety and the other phenyl ring present in these compounds (Fig. 4), to allow this ring with different options of interacting within the hydrophobic pocket where it is expected to fit. The new family of B derivatives were prepared according to Fig. 5.

The synthesis started with the coupling reaction^34^ between 1,2,4-triazole and 1-chloro-2-nitro-4-(trifluoromethyl or methyl)benzene 1a or 1b in DMF in the presence of K_2_CO_3_, to furnish bicyclic intermediates 2a^34^ and 2b^35^ in 94 and 82% yield, respectively. This reaction was first attempted on compound 4-iodobenzotrifluoride using CuI as catalyst in basic media^35^ obtaining low yield of the targeted bicyclic product. The following nitro reduction on 2a-b provided amines 3a and 3b in 51 and 68% yield, respectively.

Finally, intermediate amines 3a-b were coupled with acid derivatives 5a-c which were prepared in variable yields (60–95%) by reaction of thioglicolic acid with the corresponding phenylalkyl bromides 4a-c using NaOH as base, as previously described^33^. This coupling reaction resulted troublesome, leading to low yields of amides B1-6 when using carbodiimide based coupling reagents. Finally, the best conditions for amine 3a were achieved the corresponding acid chloride derivatives of acids 5a-c and diisopropil ethyl amine (DIPEA) as base, obtaining amides B1-3 in reasonably good yields (56–83%), whereas for amine 3b the best reaction conditions resulted using the uronium coupling reagent HATU along with carboxylic acids 5a-c and DIPEA as base, to afford the targeted compounds B4-6 in similar yields (50–83%). The structural identity of all compounds were determined by NMR spectroscopy and HRMS (see Supplementary Material).

### Binding efficiency of new molecules in vitro

**Figure 6:**
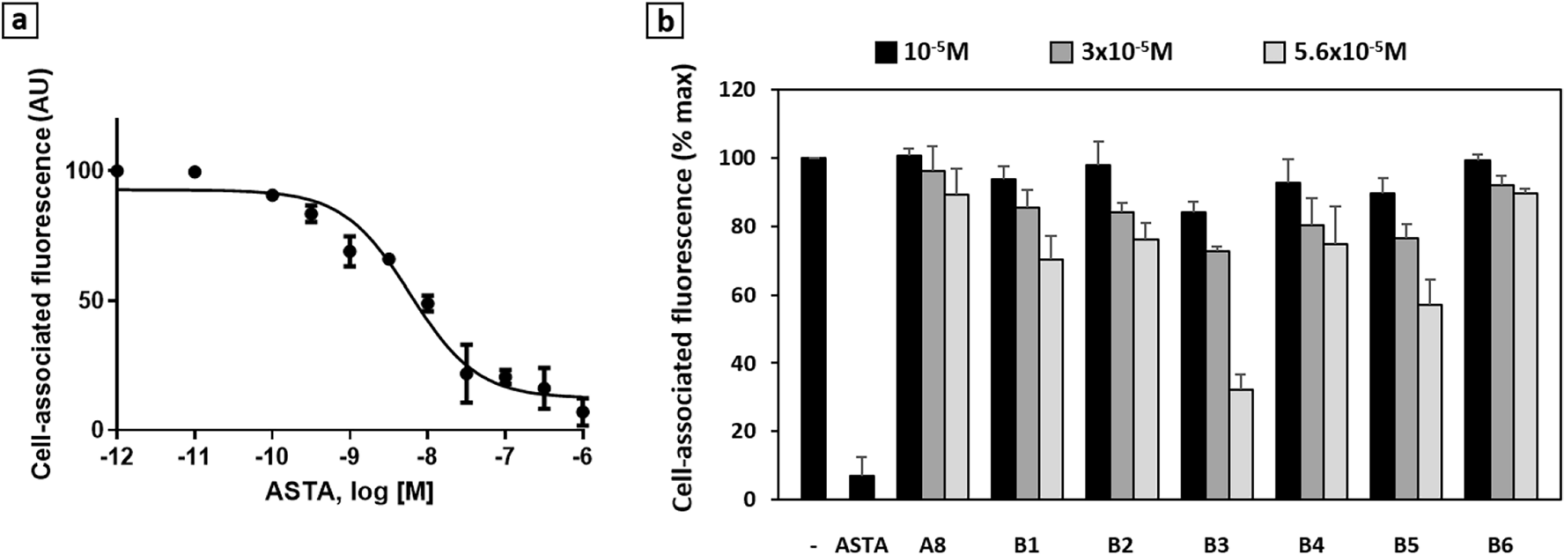
In vitro competitive binding assays for the B-series molecules. (**a**) Competition curve for the native ligand ASTA on HEK cells (4 replicates). (**b**) Results (% max) for the 6 tested B molecules, as compared with those of A8 and ASTA (10^–6^ M) (5 replicates).

The ability of these new potential ligands to bind to the ASTA receptor was tested on HEK cells, using a competitive binding assay. Their capacity to compete with fluorescence-labeled ASTA was compared to that of A8 at the same concentrations, in order to test whether the ligand had been improved by some of the chemical modifications brought to the original molecule. In all 6 cases, the affinities were low, as indicated by IC_50_ values greater than 10^–5^ M. Only B3 and B5 present affinities that seem slightly improved as compared with that of A8, but still far below that of ASTA (Figs. 6 and S3).

### Increased efficiency of new molecules as modulators of the stress response

The new selection of ligands was tested in vivo for their capacity to restore appetitive responses in stressed bees at various concentrations, using the same protocol as for compound A8. Here, the range of tested doses excluded 10^–4^ M, since we looked for significant effects that would be observable at lower doses than with A8. For the purpose of comparing the relative efficiencies of these molecules, all PBS-treated bees were pooled in a common control group, since no significant difference was found between the respective control groups (F_6,5305_ = 0.764; *p* = 0.577). The response trends over the range of sucrose concentrations can be visualized in Fig. S2. While overall, bees responded more often as sucrose got more concentrated (F_5,5305_ = 513.30; *p* < 0.001), their sensitivity was affected differently by the 6 tested molecules (F_6,1061_ = 6.11; *p* < 0.001). The proportions of responsive bees tended to increase after treatment with B2, B1 and B4, but without any obvious dose dependence. On the contrary, B3, B5 and B6 seemed to improve responsiveness to sucrose at higher doses. A repeated ANOVA on each dataset revealed a significant dose-dependent effect only for the two latter molecules (B5: F_4,340_ = 4.06; *p* = 0.003 B6: F_4,337_ = 2.75; *p* = 0.029).

**Figure 7:**
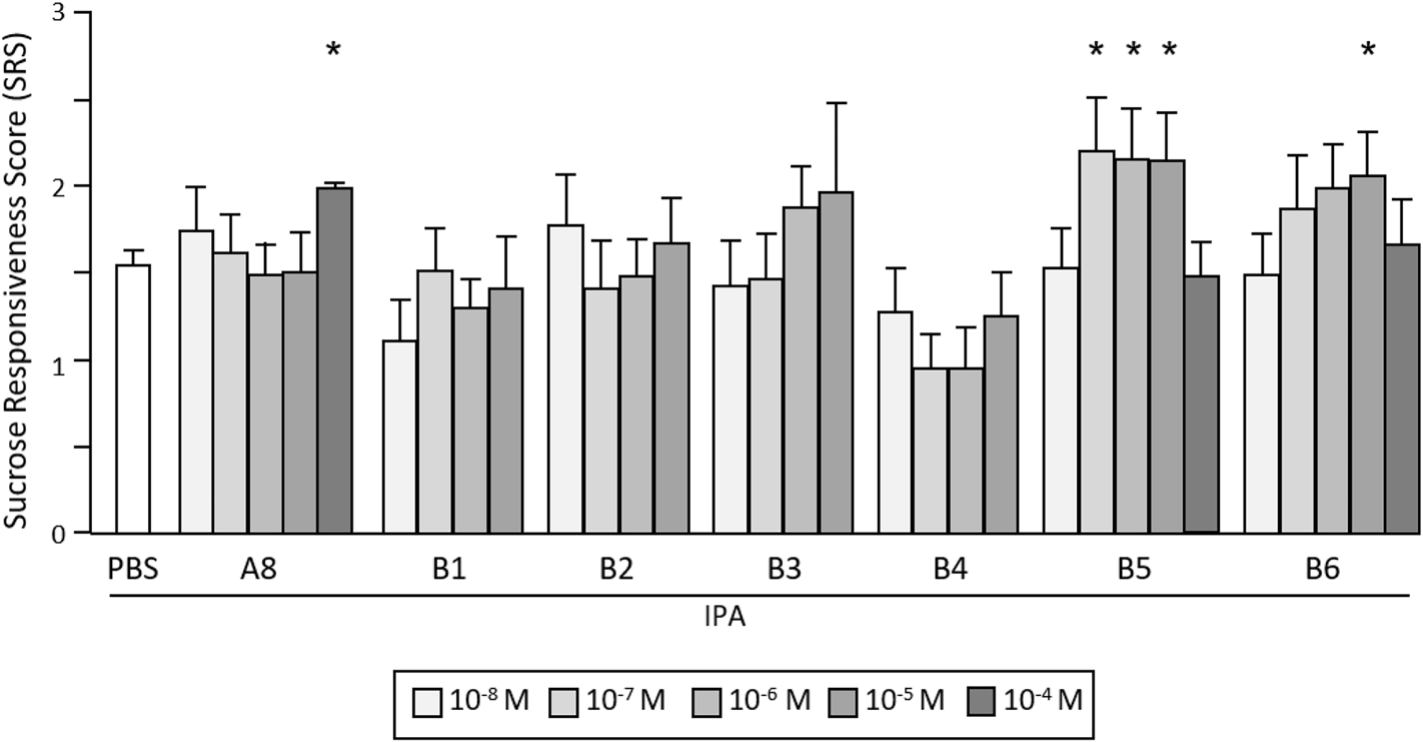
Compared effects of B-series molecules on sucrose responsiveness. Mean sucrose responsiveness score values in bees injected with PBS, A8, or either molecule of the B series (between 10^–8^ and 10^–5^ M), then exposed to IPA (2 replicates). Data from an additional experiment using injections of A8, B5 and B6 at 10^–4^ M have been included. Treatment with B5 or B6 significantly improved sucrose responsiveness, with a greater efficiency than A8, particularly for B5. *: *p* < 0.05 (PBS: n = 186; B compounds: 28–41/group).

For the purpose of comparison, we provide in Fig. 7 the mean sucrose responsiveness score values (SRS). As a mean to determine the efficient range of concentrations of B5 and B6, we ran multiple comparisons between individual doses and the PBS group. As a result, B5 induced a significant improvement of sucrose responsiveness at doses ranging from 10^–7^ to 10^–5^ M (10^–7^: F_1,240_ = 7.92; *p* = 0.005; 10^–6^: F_1,237_ = 6.76; *p* = 0.010; 10^–5^: F_1,247_ = 8.05; *p* = 0.005), and B6 did so only at 10^–5^ M (10^–5^: F_1,240=_7.46; *p* = 0.007). We further compared the effects of B5 and B6 at 10^-4^ M with those of A8 at the same concentration. The results, which have been included in Fig. 7 for the sake of comparison, show that neither B5 nor B6 could improve sucrose responsiveness at this high dose, contrary to A8 (F_1,267_ = 9.64; *p* = 0.002). Thus B5, and B6 to a lesser extent, proved efficient to reduce the effects of stress on appetitive motivation within intermediate ranges of concentrations, at lower doses than A8.

### B5 and B6 compounds act as antagonists of ASTA signaling

**Figure 8:**
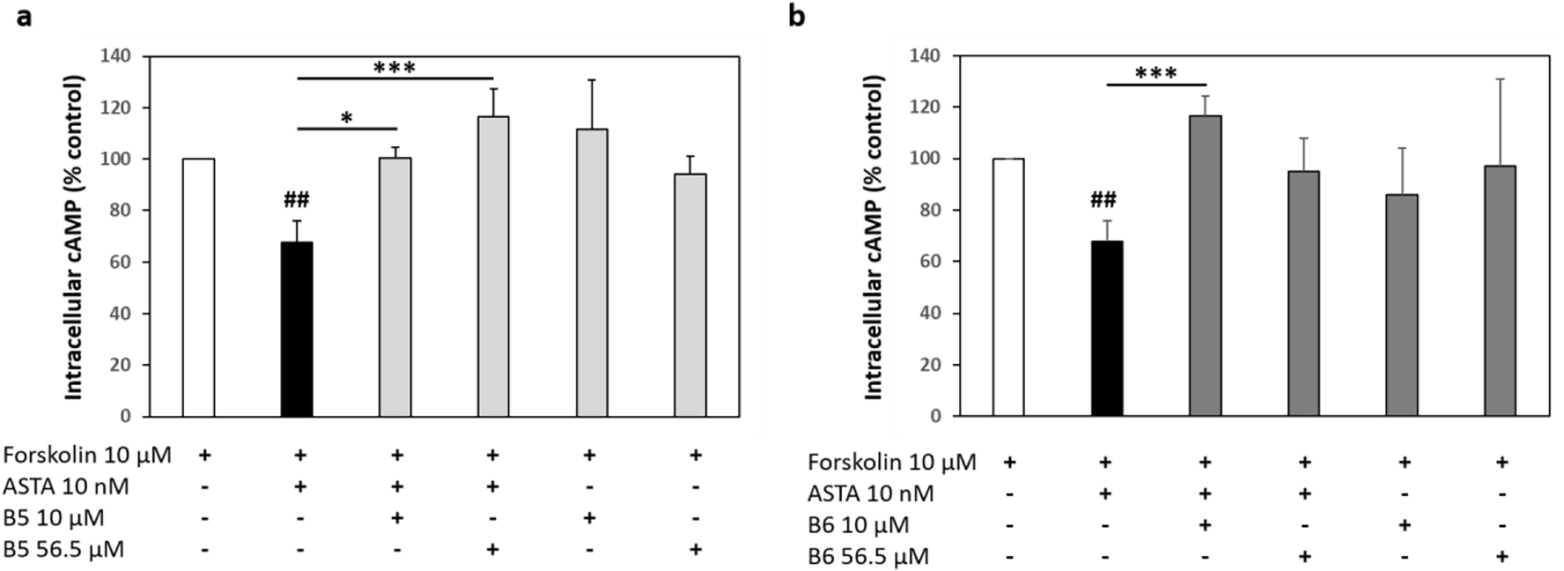
Effects of B5 and B6 compounds on ASTA signaling in vitro. Relative levels of forskolin-induced intracellular cAMP (expressed as percentages of control levels, i.e. with forskolin only). Treatment with ASTA significantly decreased cAMP levels, which was reversed when either B5 (**a**) or B6 (**b**) was added (albeit not significantly with 56.5 μM B6). ##: *p* < 0.01 (vs. forskolin only); *: *p* < 0.05; ***: *p* < 0.0005 (vs. forskolin + ASTA) (at least 5 replicates per combination).

Since B5 and B6 compounds counteract behavioural effects of stress, we hypothesized that they could do so by inhibiting the action of ASTA as a putative stress signal. Hence, we tested their capacity to modulate the action of ASTA on its receptor in transfected cells. Since the ASTA-R was shown to be coupled to the Gαi subtype^6^, which decreases cAMP (cyclic adenosine monophosphate) production via adenylyl cyclase inhibition, we exposed cells to forskolin, which activates adenylyl cyclase and increases intracellular levels of cAMP, in the presence or absence of allatostatin and/or B5 or B6 compounds. As shown in Fig. 8, ASTA significantly decreased the forskolin-induced intracellular cAMP levels by 32.2 ± 8.2% (*p* = 0.0045), indicating that, upon activation by its ligand, ASTA-R was coupled to Gαi protein and inhibited adenylyl cyclase. In presence of B5 (Fig. 8A; 10 nM: *p* = 0.013, 56.5 μM: p = 0.0002) or B6 (Fig. 8B; 10 nM: *p* = 0.0007), this capacity for ASTA to inhibit adenylyl cyclase was significantly reduced (so that the cAMP production was similar to control levels. Importantly, in absence of ASTA, neither B5 or B6 alone (in absence of ASTA) could decrease the forskolin-induced intracellular cAMP levels when incubated in ASTA-R expressing cells. These resultst suggest that both B5 and B6 act as ASTA antagonists by interacting with ASTA-R.

## Discussion

Bees are exposed to many biotic and abiotic stressors, which trigger behavioral and physiological responses through largely unknown signaling pathways. Here, we took advantage of the identified role of allatostatins in modulating behavioral responses to an ecologically-relevant stress: exposure to IPA, the main component of the sting alarm pheromone signaling potentially dangerous situations (e.g. predation). Here, a screening for potential ligands of the ASTA receptor led to identify one compound (A8) that proved efficient on behavioral measures of stress. Chemical synthesis of derivatives of this molecule enabled to increase this efficiency. To our knowledge, this is the first demonstration of a physiological effect of synthetized small-molecule ligand of a neuropeptide receptor in invertebrates. Previous work used comparable strategies to target diverse insect peptidergic pathways^36–40^ but we are unaware of published physiological or behavioral effects of the ligands identified in vitro.

Exposure to IPA triggers a series of behavioral changes that all relate to the recruitment of individuals for colony defense, on the expense of foraging: increased locomotor activity and aggressiveness^14,15^, decreased sensitivity to noxious stimuli^16,17^, reduced responsiveness to food^17,18^ and decreased performance in an appetitive learning task^18^. These behaviors are reminiscent of typical manifestations of stress in vertebrates. Injection of ASTA has similar effects on sucrose responsiveness and learning performance^6^, while it is not known yet whether ASTA recapitulates other behavioral manifestations of stress. Interestingly, in drosophila ASTA signaling seems to respond to specific physiological or environmental conditions such as nutritional stress^20–22^. Whether it participates more broadly in a more general stress response in that species is not established, but this is plausible since it promotes and coordinates the action of AKH (adipokinetic hormone) and DILPs (*Drosophila* insulin-like peptides), two signals involved in stress responses^41,42^. Here, by showing that ligands of the ASTA receptor antagonize some effects of IPA exposure, our results provide additional support for a key regulating function of allatostatins (at least ASTA) in modulating stress responses. Based on the data from *Drosophila*, ASTA might be involved in an acute stress response involving the release of glucose (and possibly lipids) in the haemolymph^21^, thus providing rapidly mobilized energy sources to fight stress such as following exposure to IPA. As a consequence, bees would behave as if satiated, i.e. displaying low appetitive motivation for sucrose and decreased performance in appetitive learning. Consistently with this model, the capacity of the tested compounds to promote sucrose responsiveness and (at least for A8) re-establish learning performance in stressed bees, might be the consequence of limiting IPA-triggered glucose release. Since such molecules here could reverse the effects of IPA exposure, they appear to counteract that of ASTA. As they could also compete with ASTA in binding assays and inhibit its signaling through adenylyl cyclase, they appear to act as competitive antagonists of the ASTA receptor. Yet, their selectivity remains to be assessed, so that we cannot exclude that they act on the ASTC receptor and/or others, particularly given their relatively high concentration range of action.

Independently of their exact mechanisms of action, some of the compounds tested in this study could modulate different behavioral manifestations of stress, in a dose-dependent manner. In particular, the A8 compound proved to restore, at least partially, both the learning performance and the motivation for sucrose. Since the learning task is a standard appetitive assay using sucrose as a reward, in principle the effects on learning performance might be a mere consequence of changes in appetitive motivation (the higher the motivation, the higher the probability to learn). Yet, both actions appear to be largely independent. First, learning was assessed only in bees responding to 50% sucrose, thus ensuring a sufficient motivation in all trained bees. It was simply the capacity to reach this minimal level of motivation (before learning) that was modulated by A8. Second, when A8 significantly affected responsiveness to less concentrated sucrose, it did so by increasing it, which would rather predict decreased learning (bees with higher SRS values tend to learn less well, as their motivation is lower^43,44^. Thus, A8 modulated learning performance in responding bees independently of its reducing action on responsiveness scores. Importantly, this dual effect is reminiscent of the situation in fruitflies, where blocking ASTA-expressing neurons could impair sugar-mediated appetitive learning without affecting innate sugar preference^23^. However, a notable difference between the two species is that ASTA decreases sucrose responsiveness but promotes appetitive learning in flies, while it reduced both in bees^19^. Nevertheless, the effects of A8 thus mirror those triggered by IPA exposure^18^; however, they were obtained in the millimolar range (10^–4^ M), while ASTA could significantly impair learning already in the micromolar range (10^–6^–10^–4^ M)^6^. This limited efficiency advocated for the search of more efficient compounds.

As an assessment of in vivo effects of the chemically-synthetized derivatives of A8 (B molecules), we focused on SRS measurement as a rather quick way to screen several compounds at multiple doses. This approach enabled revealing a capacity for B5 and B6 to increase sucrose responsiveness, thus having more potent modulatory effects on appetitive motivation than A8. In addition, they displayed such biological activity at concentrations respectively 1000 and 10 times lower than for A8, thus in a range comparable to that of ASTA^6^. They thus appear as more potent modulators on the basis of this preliminary work. While further experiments will be necessary in the future to assess their effects on other behavioral parameters of the stress response, they are clearly (particularly B5) interesting candidate modulators of the bee stress response, through the inhibition of ASTA signaling. Which structural changes may explain this improvement? The substitution of the –CF_3_ group on the phenyl ring, a common point between B5 and B6, appears to be a major factor of improvement, since no behavioral effect was detected using any fluorinated derivative (B1-3). In addition, a length of 2–3 carbon atoms for the chain connecting the phenyl rings seems optimal, since with a shorter chain B4 displayed no clear effect. Interestingly, B3, with a 3-carbon chain, was the only fluorinated compound showing a trend for a dose-dependent improvement of sucrose responsiveness. Likewise, only B3 and B5 had a slightly increased affinity for the receptor in our in vitro binding assay. However, these values remained low, and the substitution of the –CF_3_ group had no clear effect on affinity, thus making difficult to relate chemical specificities, binding properties and physiological effects. There are several possible explanations for this discrepancy. The actual conformation of the receptor in vivo might be more favorable towards B5 binding, or the de-fluorinated compounds might show improved tissue penetration (and thus efficiency) due to a reduced hydrophobicity^40,45^. Such hypotheses might also explain why A16 or A23 (which bears a fluorine group) failed to modulate learning in preliminary experiments when used as the same concentration as A8, despite rather similar affinities in vitro, although we cannot discard that they might be effective at different doses and/or might affect other behavioral parameters.

Earlier work suggested that the activation of ASTA-R might reduce cyclic AMP levels through coupling with Gi (consistently with our results own results) and/or Go proteins^6^, as for its closest mammalian homologue, GALR1^46,47^. However, the protective effects of galanin under stressful conditions are due to its capacity to inhibit the hypothalamic–pituitary–adrenal through GALR1^48^, while ASTA rather promotes stress-like responses. Thus, it seems that signaling through ASTA and galanin receptors modulate stress in opposite directions. Consistently, as discussed above the compounds appear to modulate sucrose responsiveness by acting as antagonists, while in mammals it is the activation of GAL receptors (most likely GALR1) by galanin that attenuates the activity of midbrain reward circuits^24^. Thus, despite conservation of some of their structural and functional features, ASTA and GAL receptors appear to modulate stress responses through different mechanisms. This conclusion supports the necessity to further explore the specificities of stress regulation in bees, and to develop specific strategies to improve their resilience. The present work is a first step in this novel direction, in contrast with most studies searching for ligands of insect receptors for pest control purposes [e.g.^38,39^], and calls for further work to investigate whether the effects of other stressors on bee learning and/or sucrose responsiveness (e.g. parasites, pesticides^48–51^) are mediated by the allatostatin system. Nevertheless, the present study shows that the search for synthetic compounds targeting specific bee receptors can prove effective, a strategy that can be extended to target other pathways as well.

## Supplementary Information

Supplementary Information.

## Data Availability

The datasets generated and analyzed during the current study are available from the corresponding authors on reasonable request.
