# Supplementary material for: Reduction of stress responses in honey bees by synthetic ligands targeting an allatostatin receptor
Source: Sci Rep. 2022 Oct 6;12:16760. doi: 10.1038/s41598-022-20978-y (PMC9537510; doi:10.1038/s41598-022-20978-y)
Supplement: Supplementary file 1 — Supplementary Information. [file 41598_2022_20978_MOESM1_ESM.pdf]

Supplementary Table 1: List of compounds (A1-A23) selected for *in vitro* binding experiments, based on the virtual screening for ligands of the ASTA receptor.

Molecule	ZINC Nbr.	MolPort Ref. Nbr.	Provider – Catalog Nbr.
**A1**	08961583	003-113-947	Life Chemicals Inc. - F2277-0085
**A2**	00343628	002-799-596	Specs - AC-907/34130057
**A3**	06938450	004-093-769	ENAMINE Ltd. - Z45611413
**A4**	60362419	010-428-507	ENAMINE Ltd. - Z1020952700
**A5**	04957299	002-136-046	ChemBridge Corporation - 5211388
**A6**	12787609	005-668-019	ENAMINE Ltd. - Z30141626
**A7**	65194116	010-912-878	ChemDiv, Inc. - M952-1127
**A8**	22617467	006-045-762	ENAMINE Ltd. - Z24861695
**A9**	00246283	000-162-802	ChemBridge Corporation - 5316696
**A19**	13894175	002-943-804	Vitas-M Laboratory, Ltd. - STK087888
**A11**	13183644	005-454-560	ENAMINE Ltd. - Z85907627
**A12**	04276367	002-247-882	Life Chemicals Inc. - F1407-0215
**A13**	35397617	010-737-204	ChemDiv, Inc. - F293-0690
**A14**	69622155	020-019-052	ENAMINE Ltd. - Z1138601758
**A15**	67641273	019-798-932	ChemBridge Corporation - 10257373
**A16**	02236226	000-830-195	InterBioScreen Ltd. - STOCK3S-72356
**A17**	13186976	005-458-297	ENAMINE Ltd. - Z85969560
**A18**	63211889	002-010-141	ENAMINE Ltd. - Z56971771
**A19**	08754917	003-097-856	ENAMINE Ltd. - Z237523122
**A20**	04868082	002-101-186	ChemBridge Corporation - 9007792
**A21**	15774319	007-850-500	ChemDiv, Inc. - BB01-3683
**A22**	00870369	001-908-122	Vitas-M Laboratory, Ltd. - STK077856
**A23**	12997949	009-624-345	ENAMINE Ltd. - Z238539738
**A24**	01282655	000-696-223	Vitas-M Laboratory, Ltd. - STK541026

^{1}H and ^{13}C NMR spectra

	page
^{1}H NMR spectrum of compound **5a**	2
^{1}H NMR spectrum of compound **5b**	2
^{1}H NMR spectrum of compound **5c**	3
^{1}H and ^{13}C NMR spectra of compound **2a**	4
^{1}H and ^{13}C NMR spectra of compound **2b**	5
^{1}H and ^{13}C NMR spectra of compound **3a**	6
^{1}H and ^{13}C NMR spectra of compound **3b**	7
^{1}H and ^{13}C NMR spectra of compound **B1**	8
^{1}H and ^{13}C NMR spectra of compound **B2**	9
^{1}H and ^{13}C NMR spectra of compound **B3**	10
^{1}H and ^{13}C NMR spectra of compound **B4**	11
^{1}H and ^{13}C NMR spectra of compound **B5**	12
^{1}H and ^{13}C NMR spectra of compound **B6**	13

^{1}**H NMR** (360 MHz, DMSO-d$_6$)**, 5a**

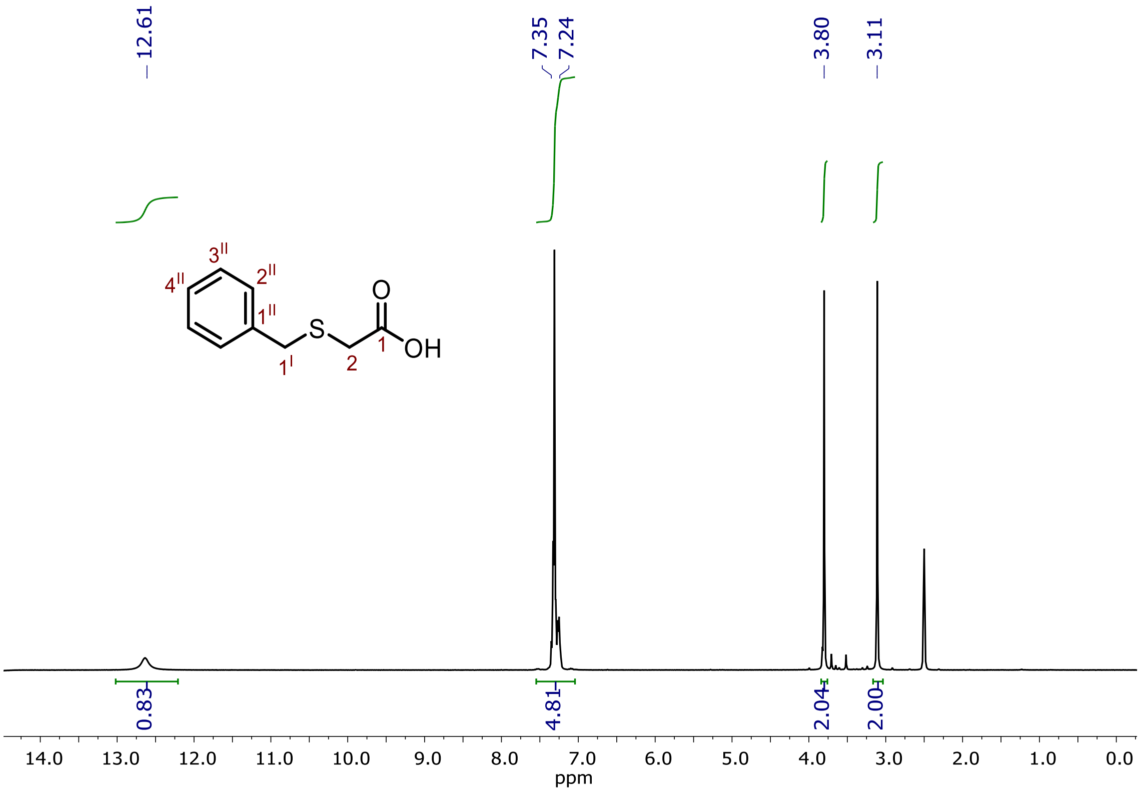

^{1}**H NMR** (360 MHz, DMSO-d$_6$)**, 5b**

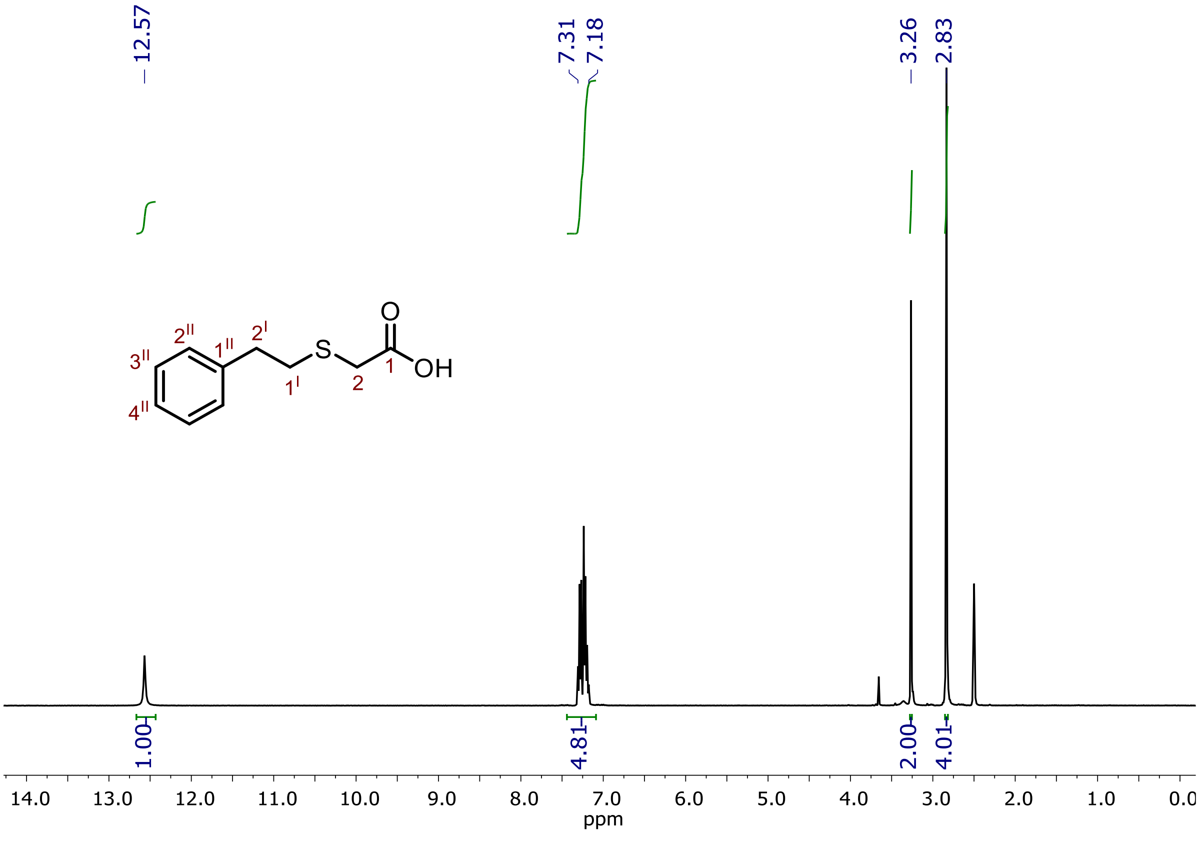

^{1}H NMR (360 MHz, DMSO-d_6)**, 5c**

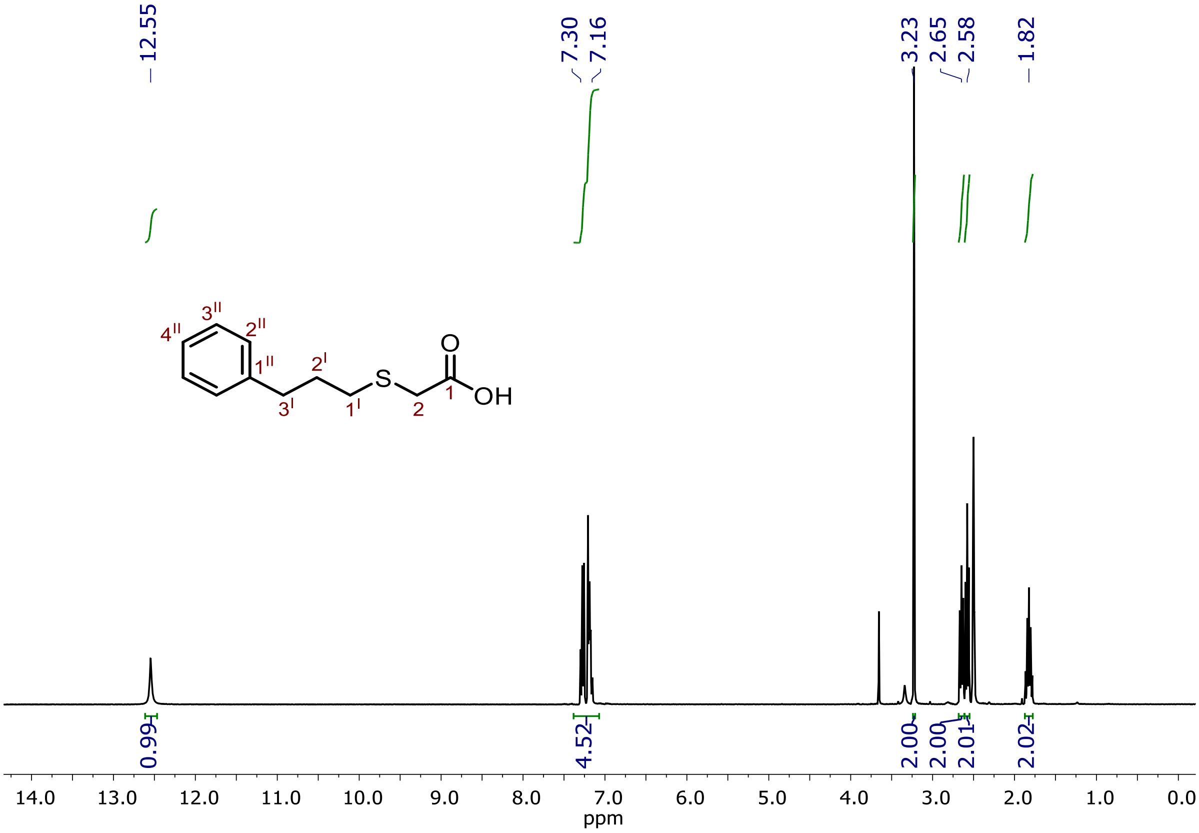

^{1}H NMR (360 MHz, CD_2Cl_2)**, 2a**

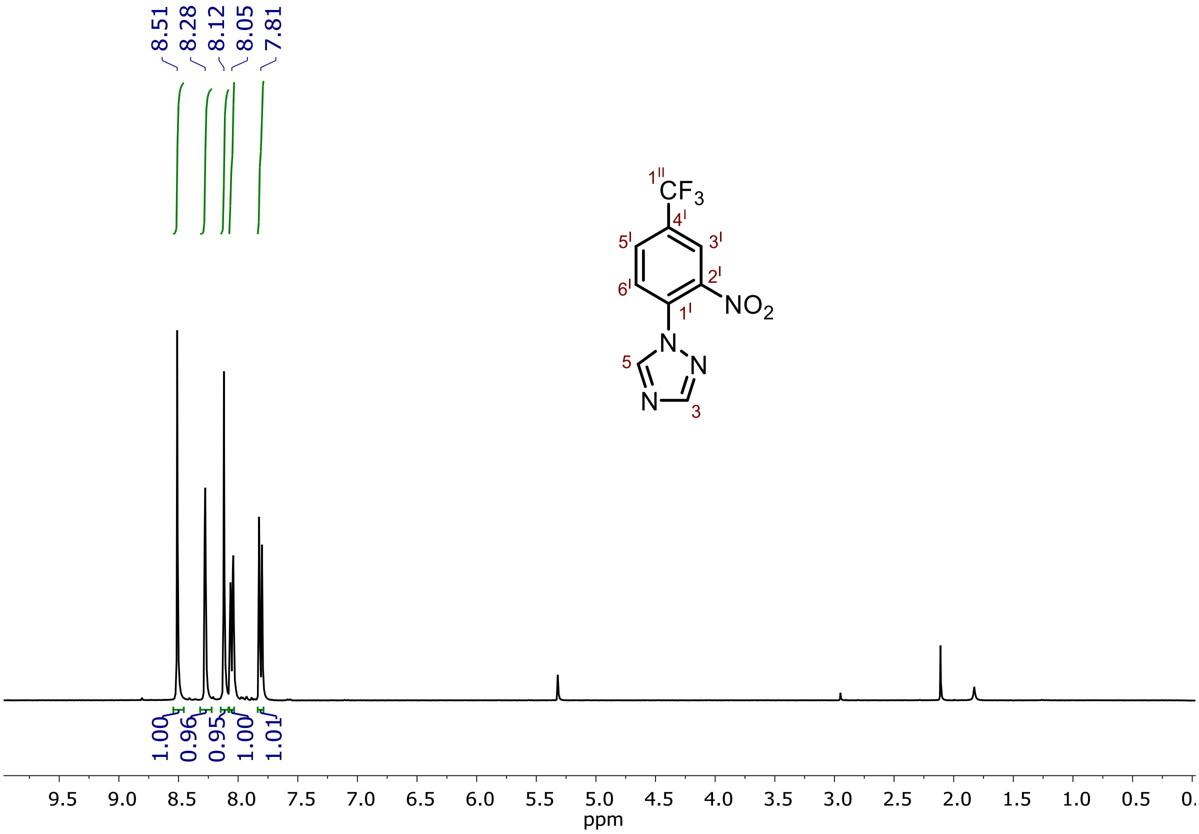

^{13}C NMR (90.5 MHz, CD_2Cl_2), **2a**

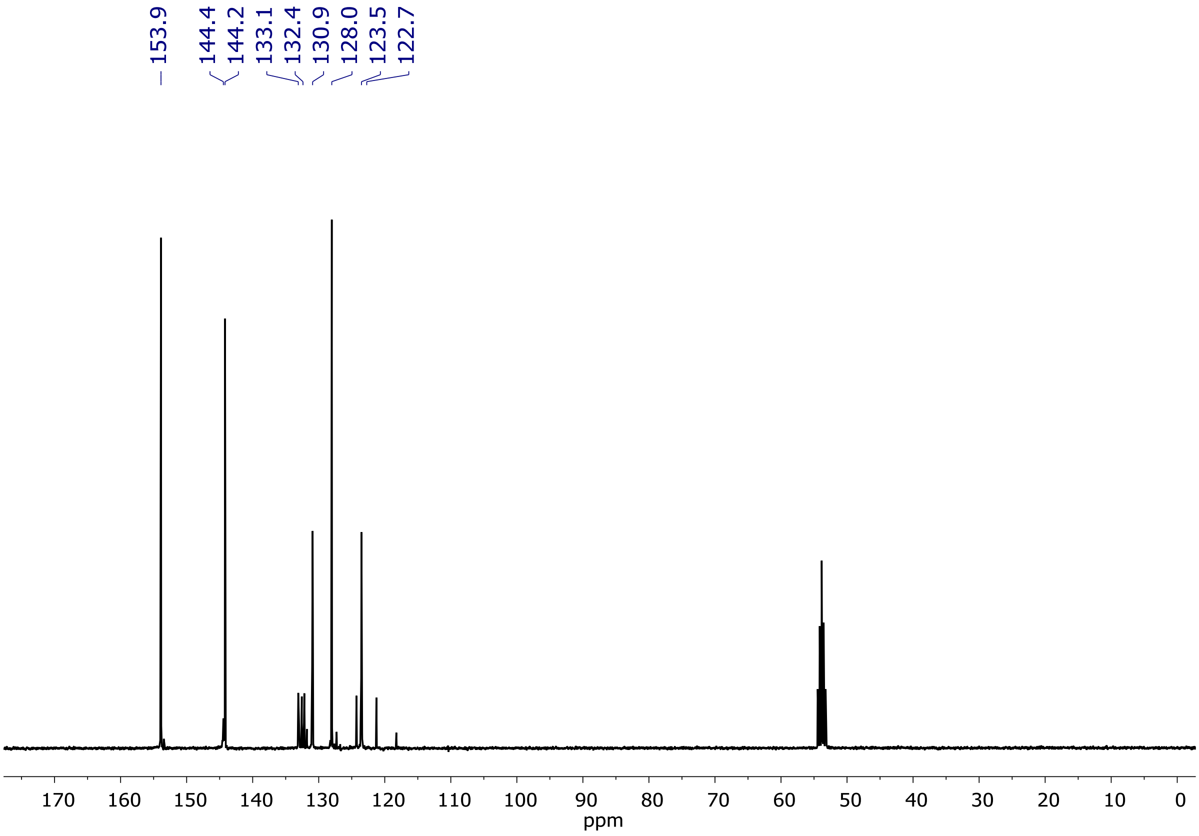

^{1}H NMR (400 MHz, $CDCl_3$)**, 2b**

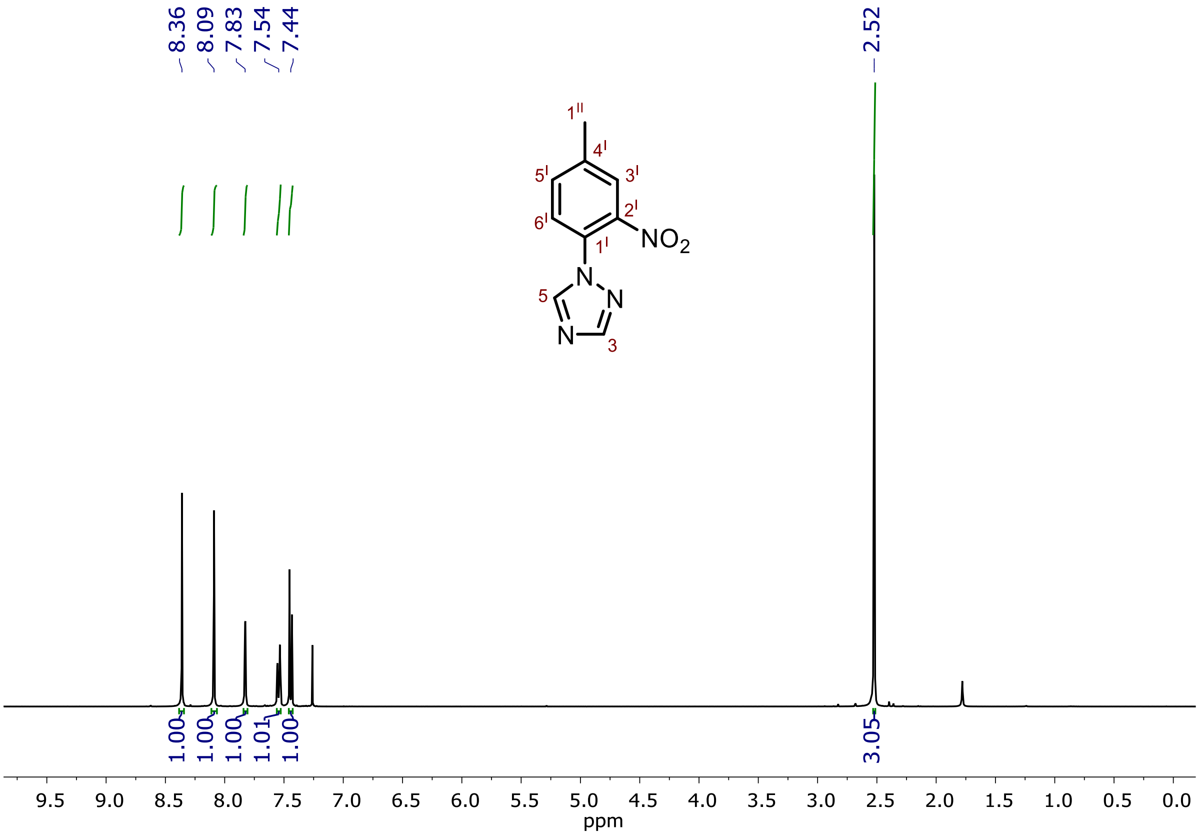

^{13}C NMR (100.6 MHz, $CDCl_3$), **2b**

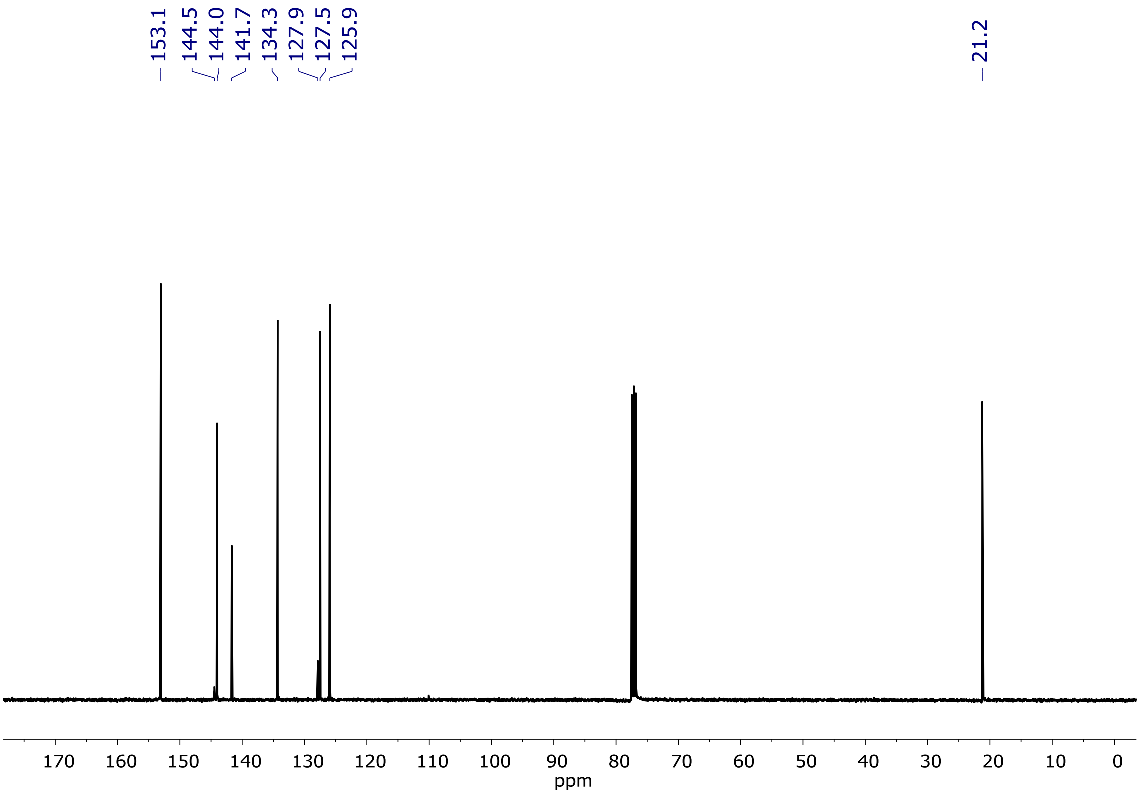

^{1}H NMR (360 MHz, CD_2Cl_2)**, 3a**

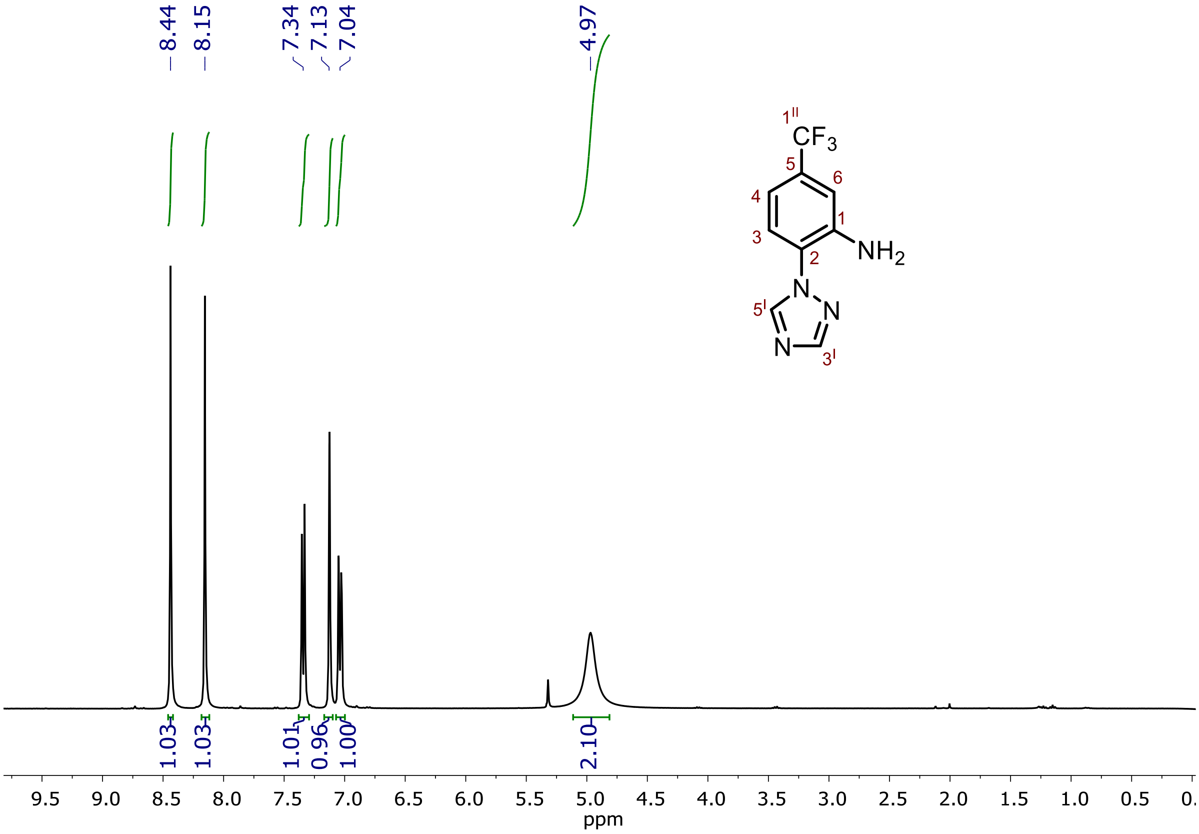

^{13}C NMR (90.5 MHz, CD_2Cl_2), **3a**

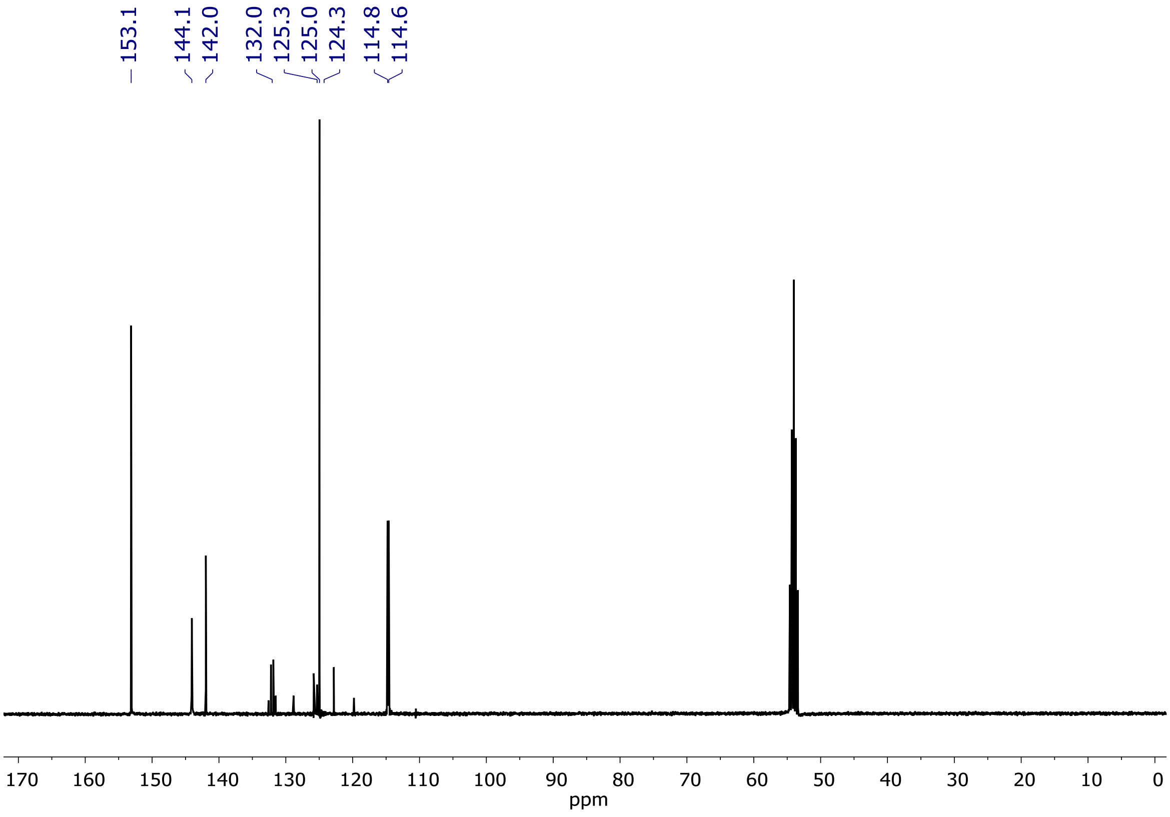

^{1}H NMR (400 MHz, $CDCl_3$)**, 3b**

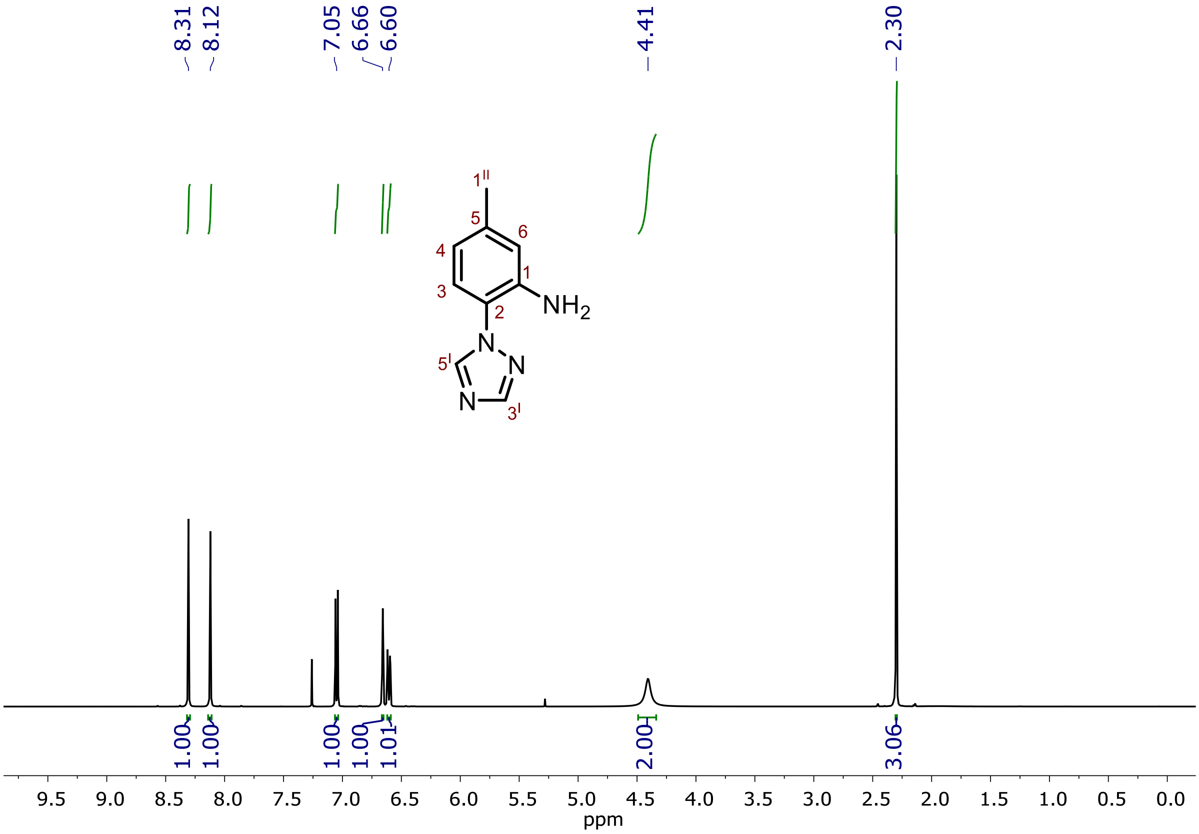

^{13}C NMR (100.6 MHz, $CDCl_3$), **3b**

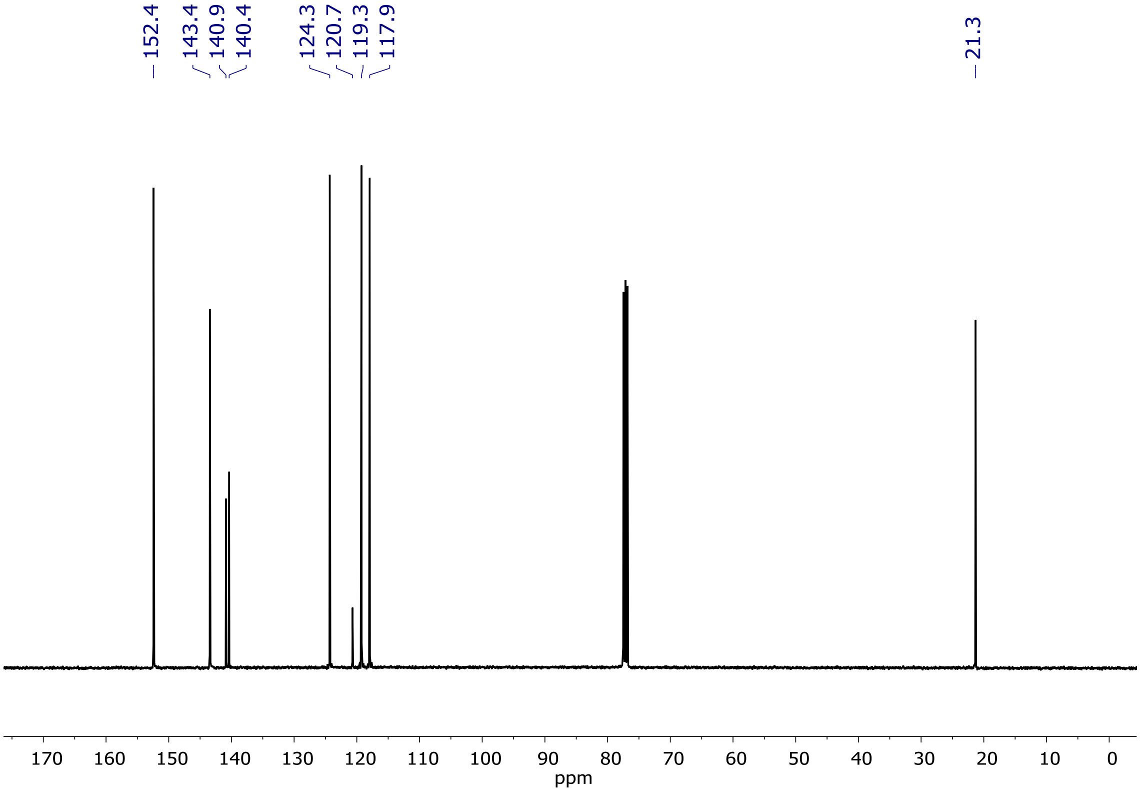

^{1}H NMR (360 MHz, CD_2Cl_2)**, B1**

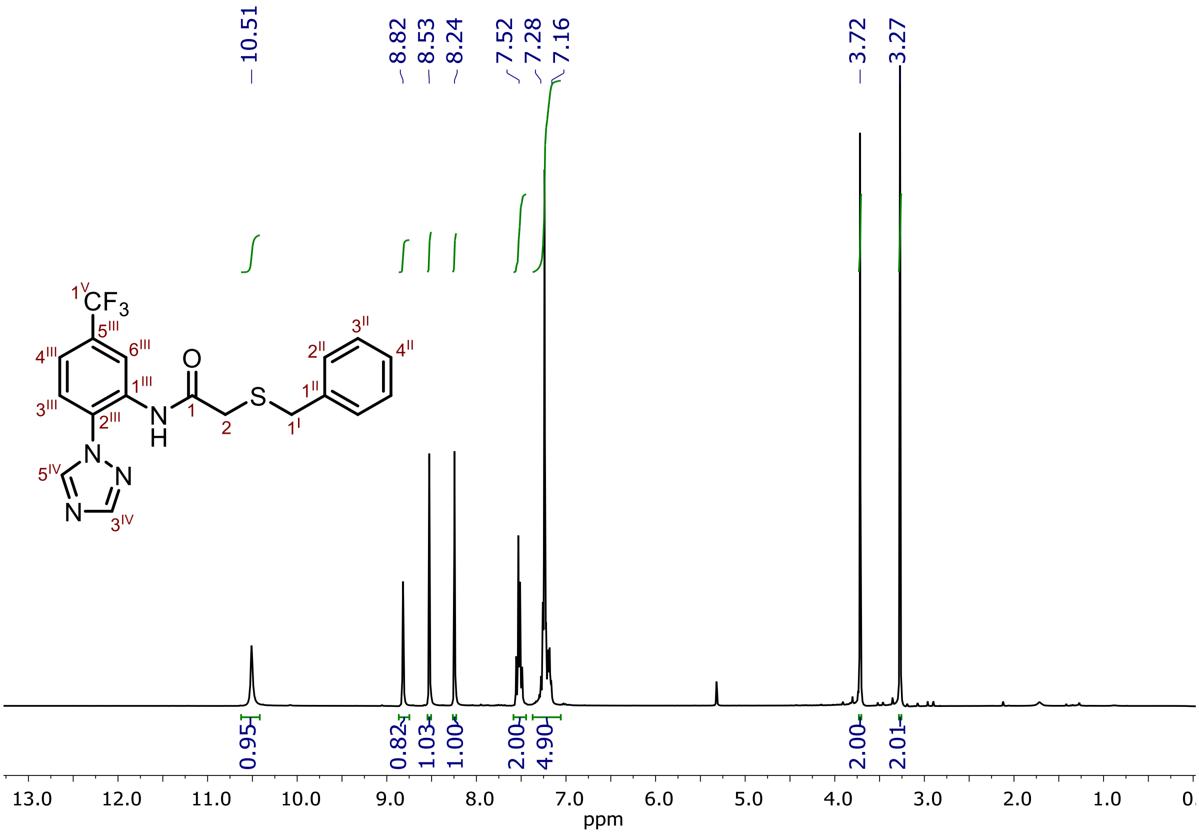

^{13}C NMR (90.5 MHz, CD_2Cl_2), **B1**

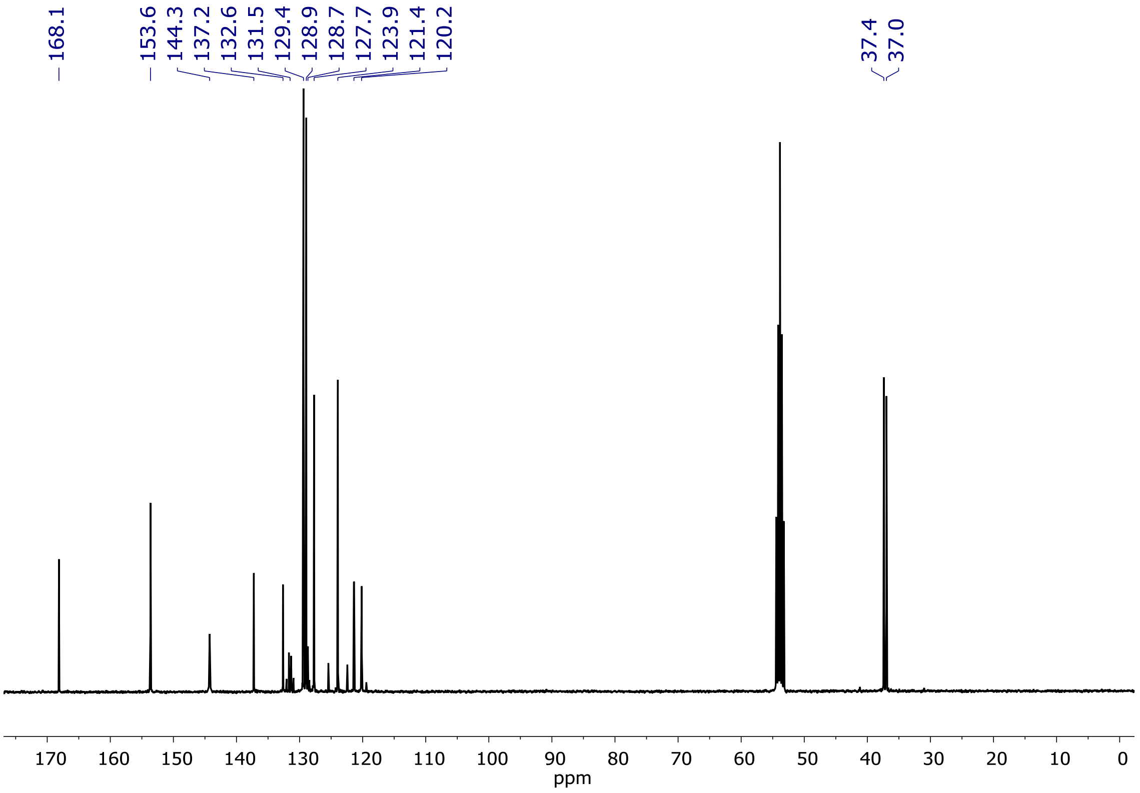

^{1}H NMR (400 MHz, CD_2Cl_2)**, B2**

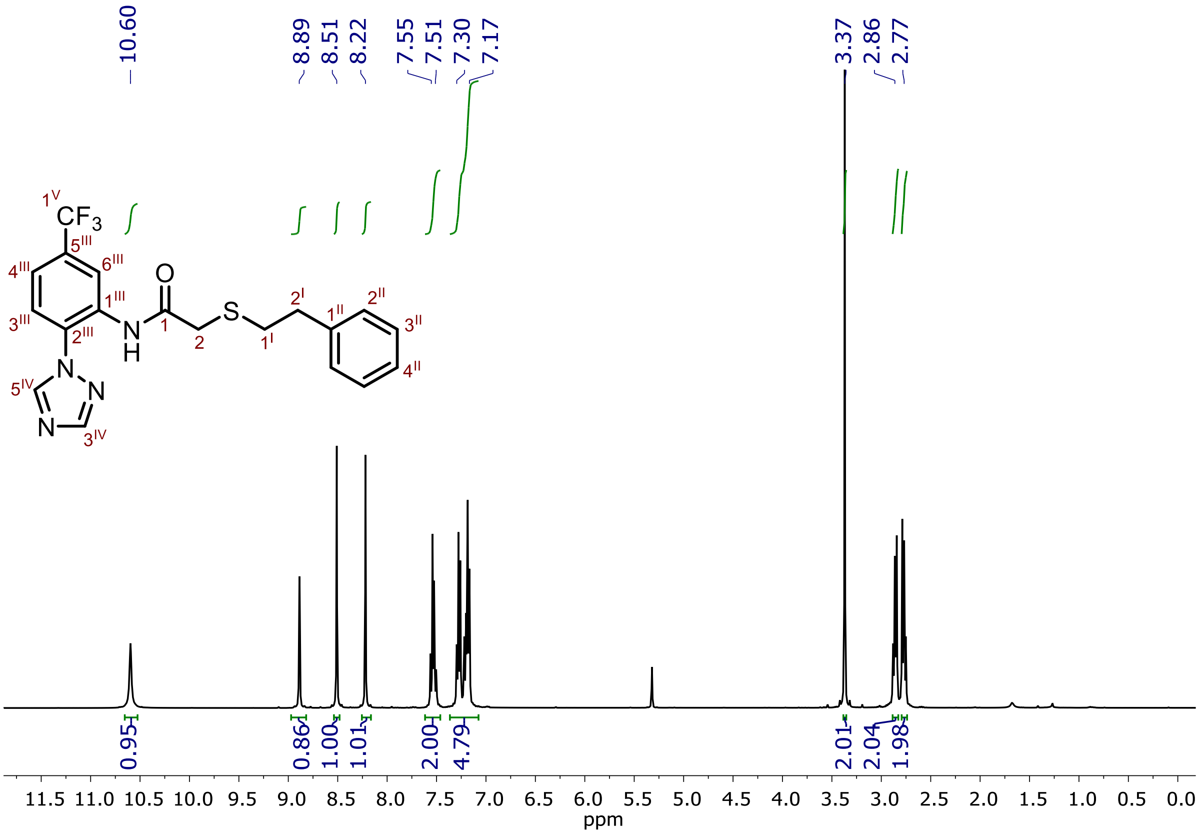

^{13}C NMR (100.6 MHz, CD_2Cl_2), **B2**

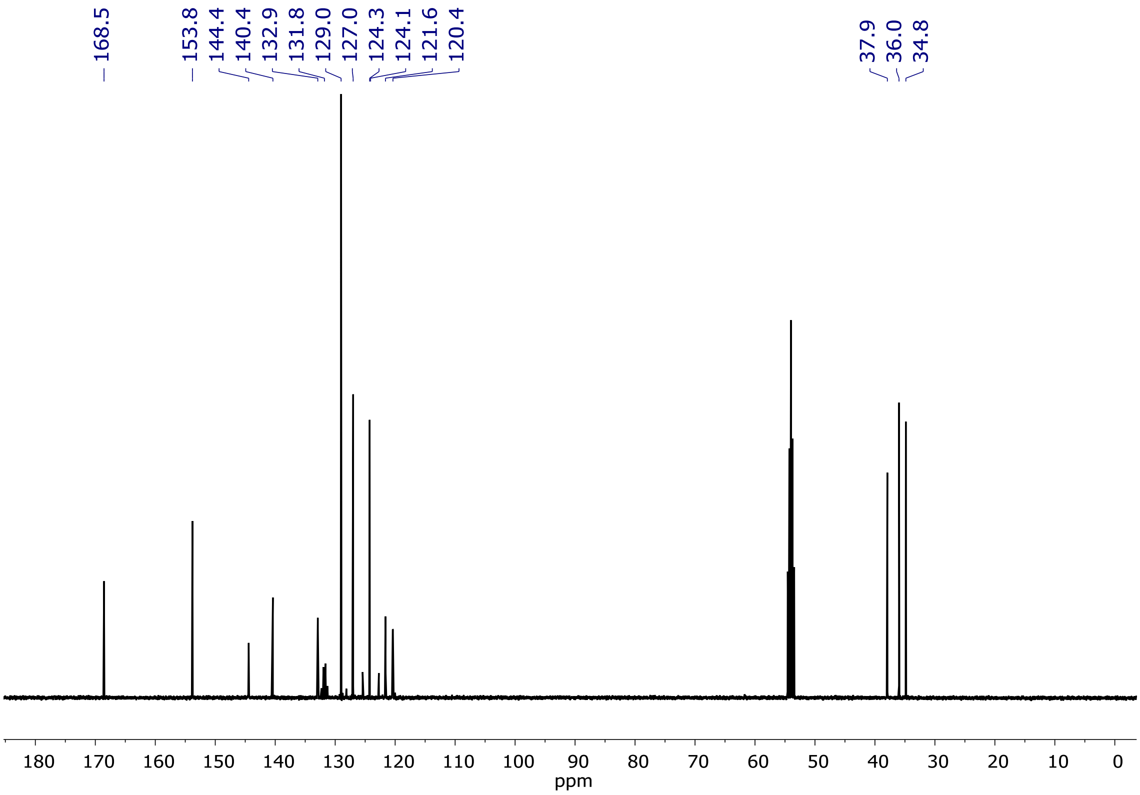

^{1}H NMR (400 MHz, CD_2Cl_2)**, B3**

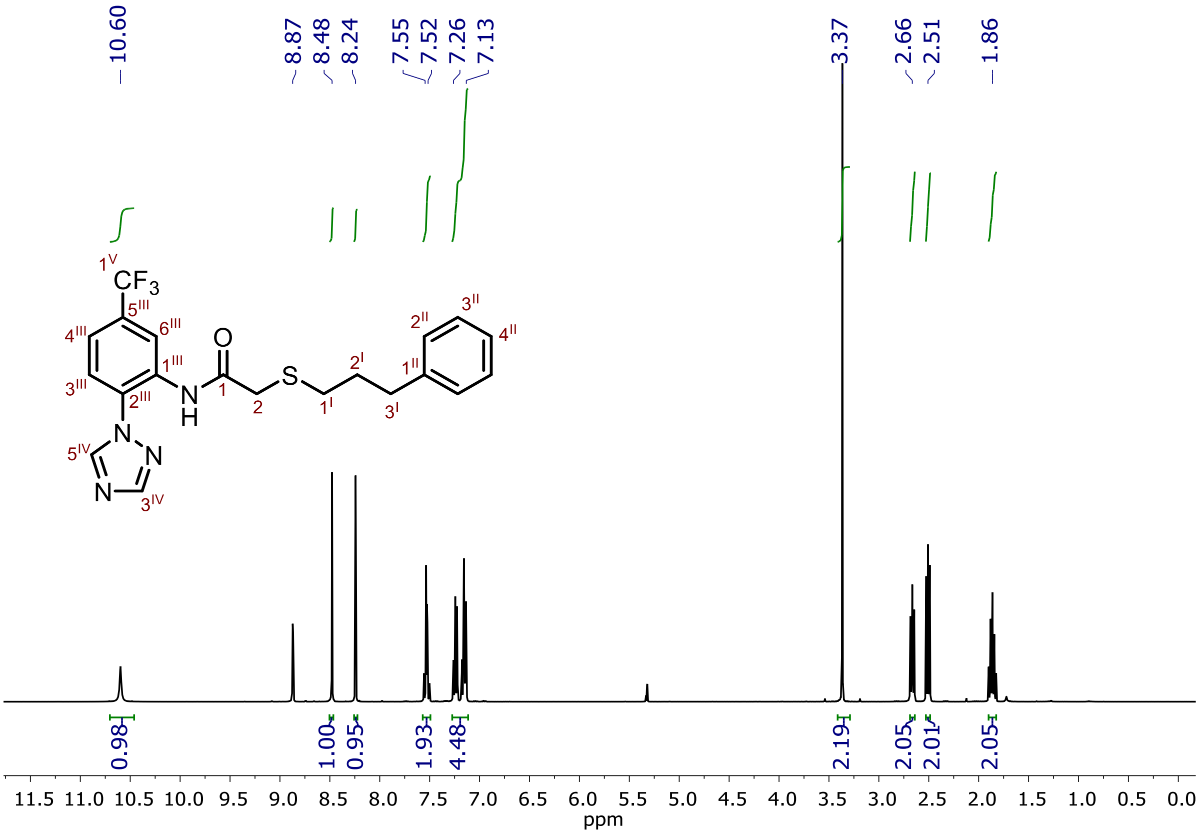

^{13}C NMR (100.6 MHz, CD_2Cl_2), **B3**

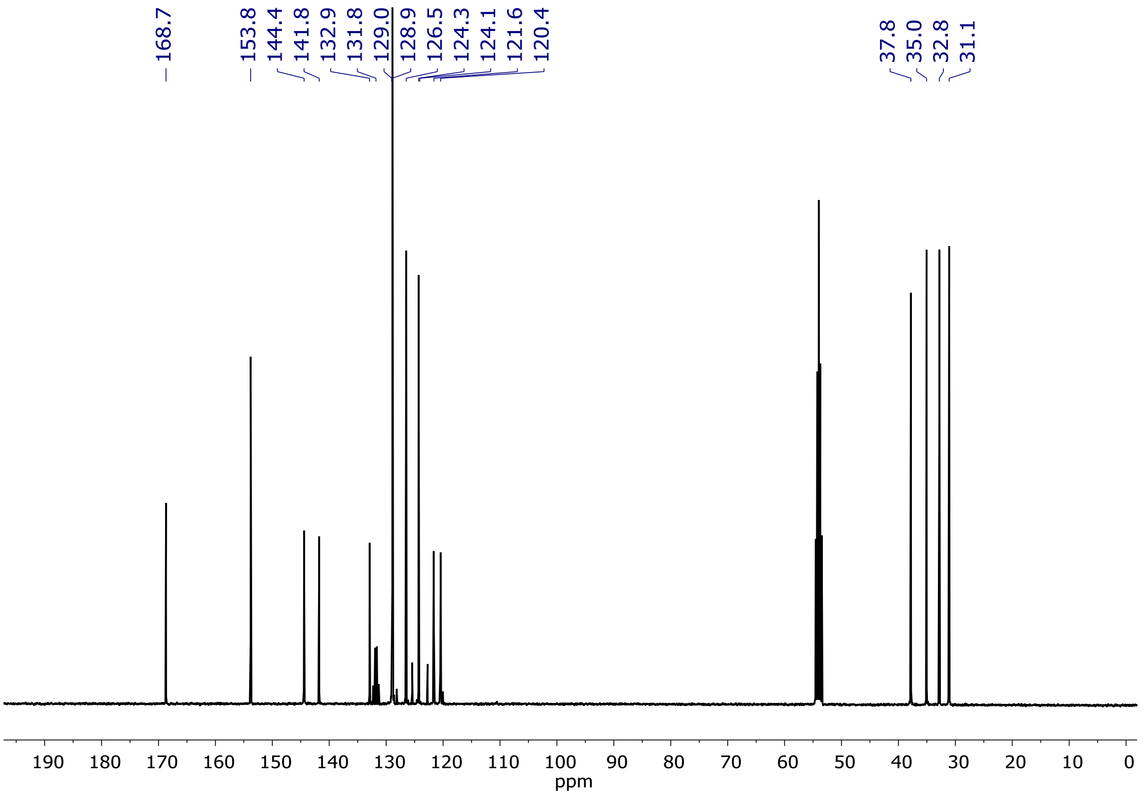

^{1}H NMR (400 MHz, acetone-d_6)**, B4**

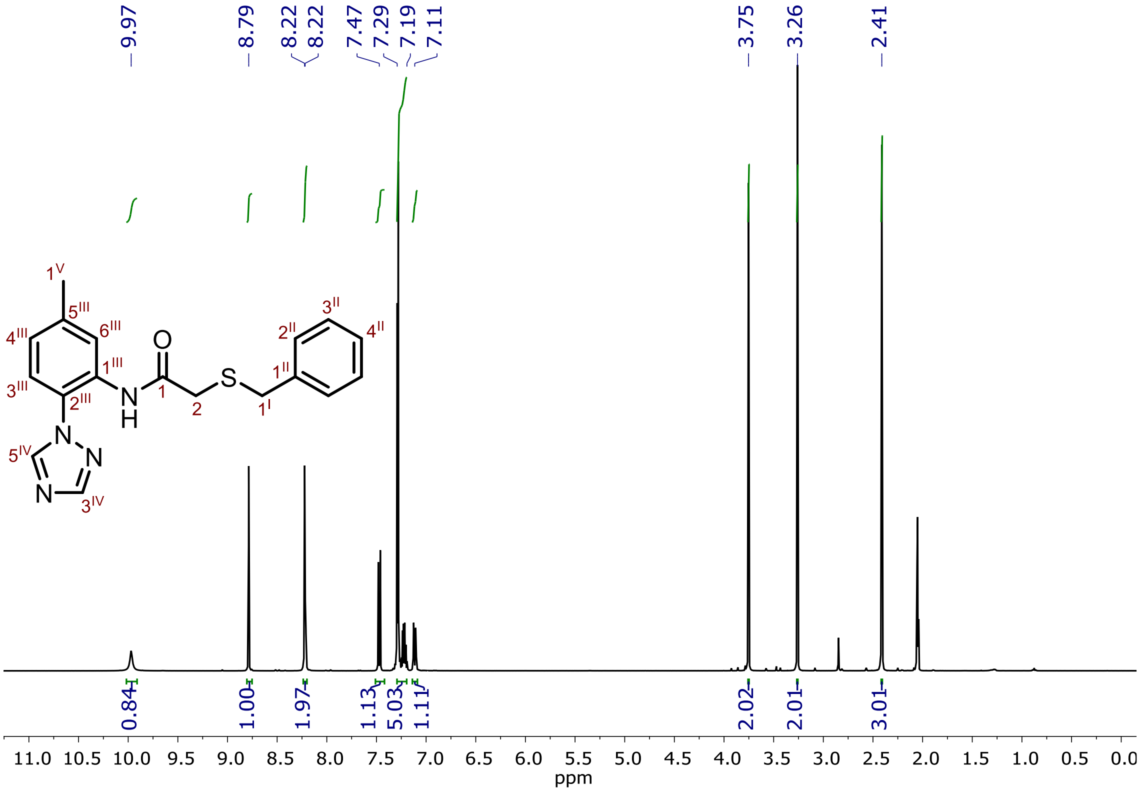

^{13}C NMR (100.6 MHz, acetone-d_6), **B4**

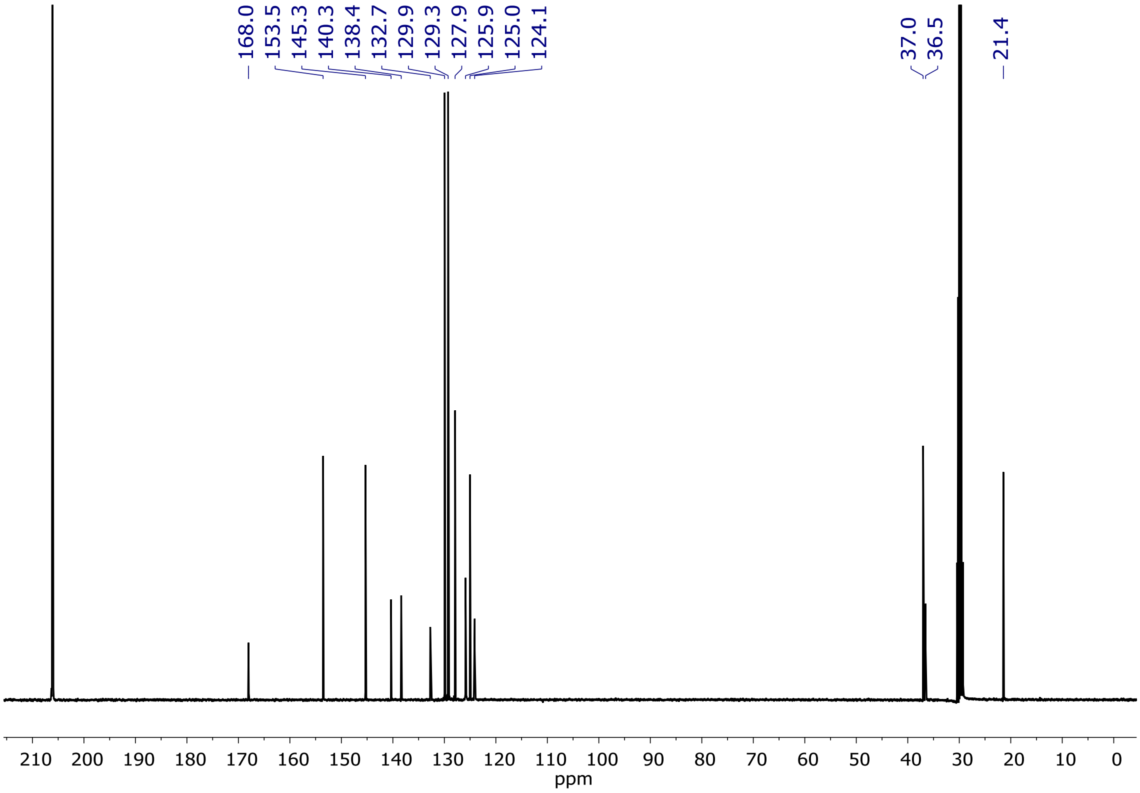

^{1}H NMR (360 MHz, CD_2Cl_2), **B5**

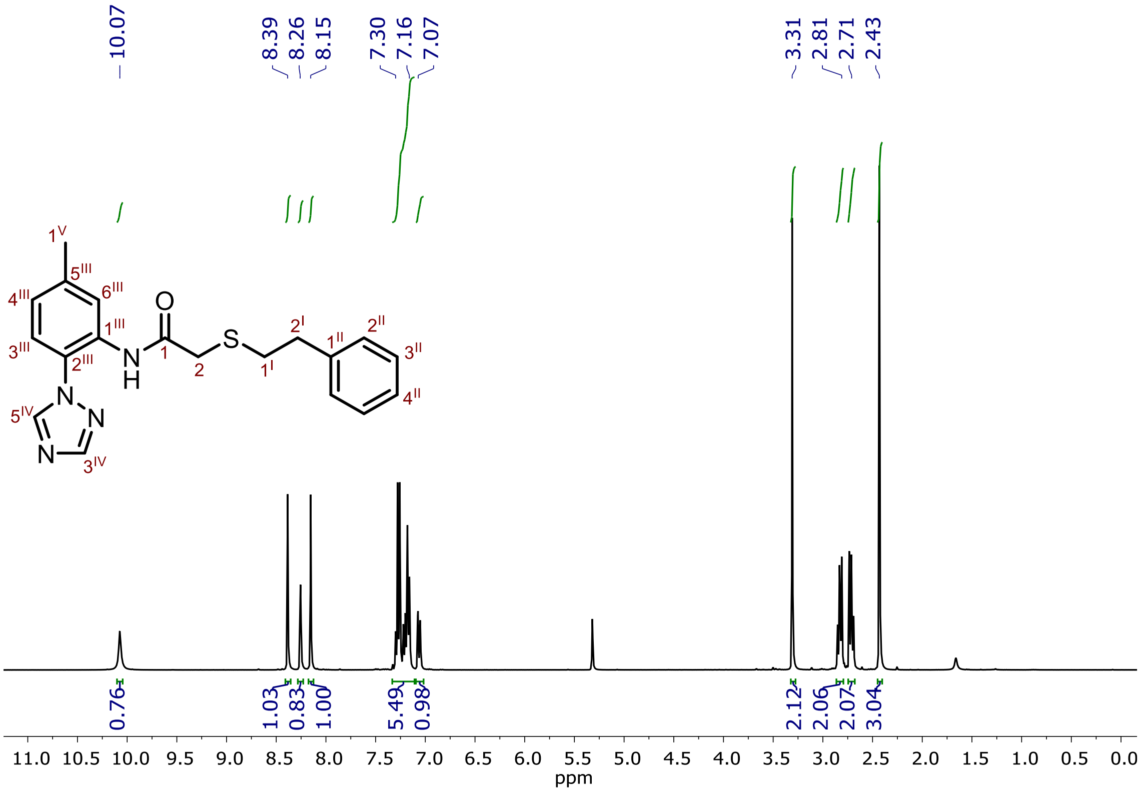

^{13}C NMR (90.5 MHz, CD_2Cl_2), **B5**

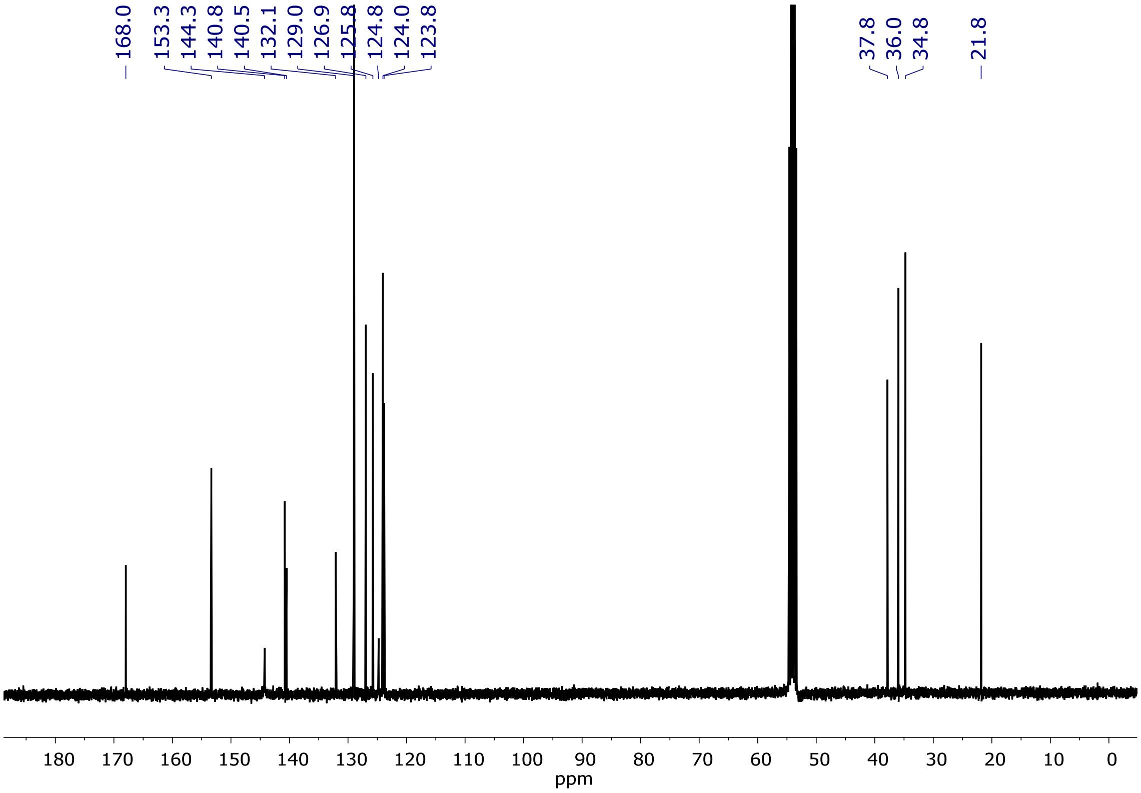

^{1}H NMR (400 MHz, CD_2Cl_2)**, B6**

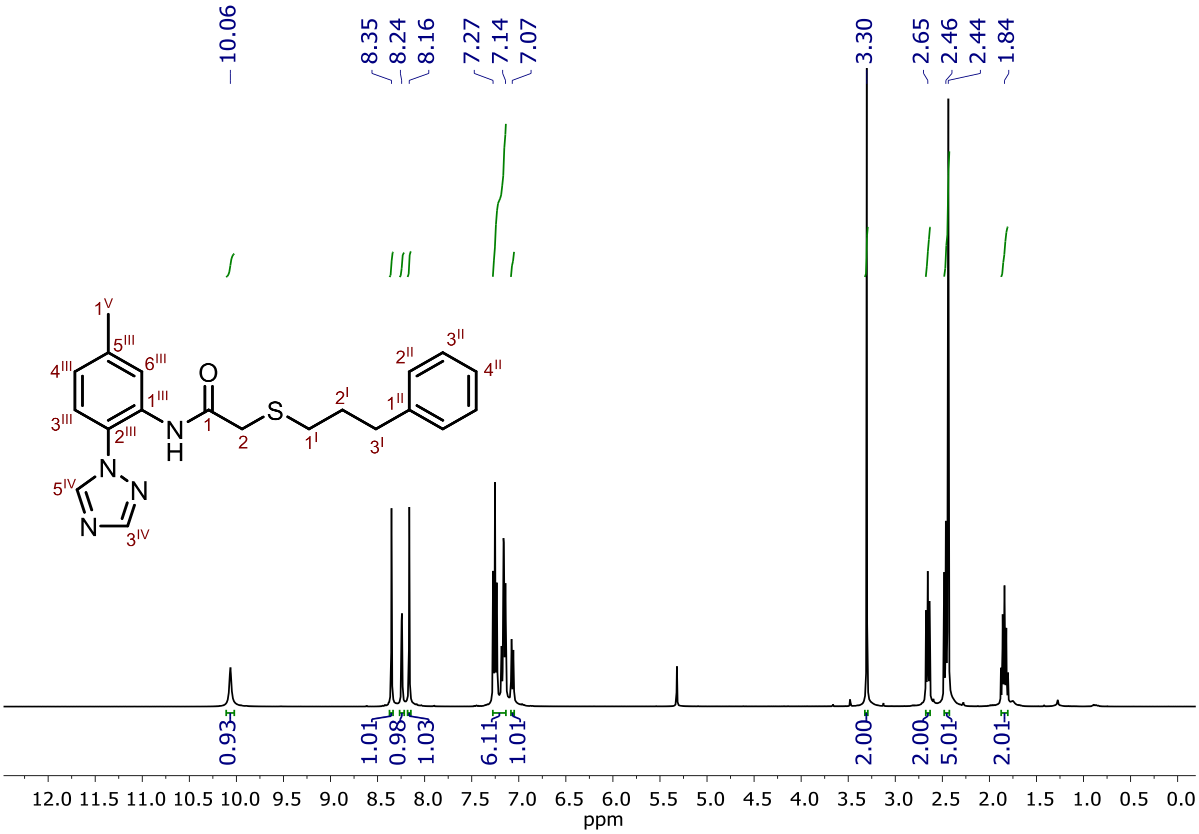

^{13}C NMR (100.6 MHz, CD_2Cl_2), **B6**

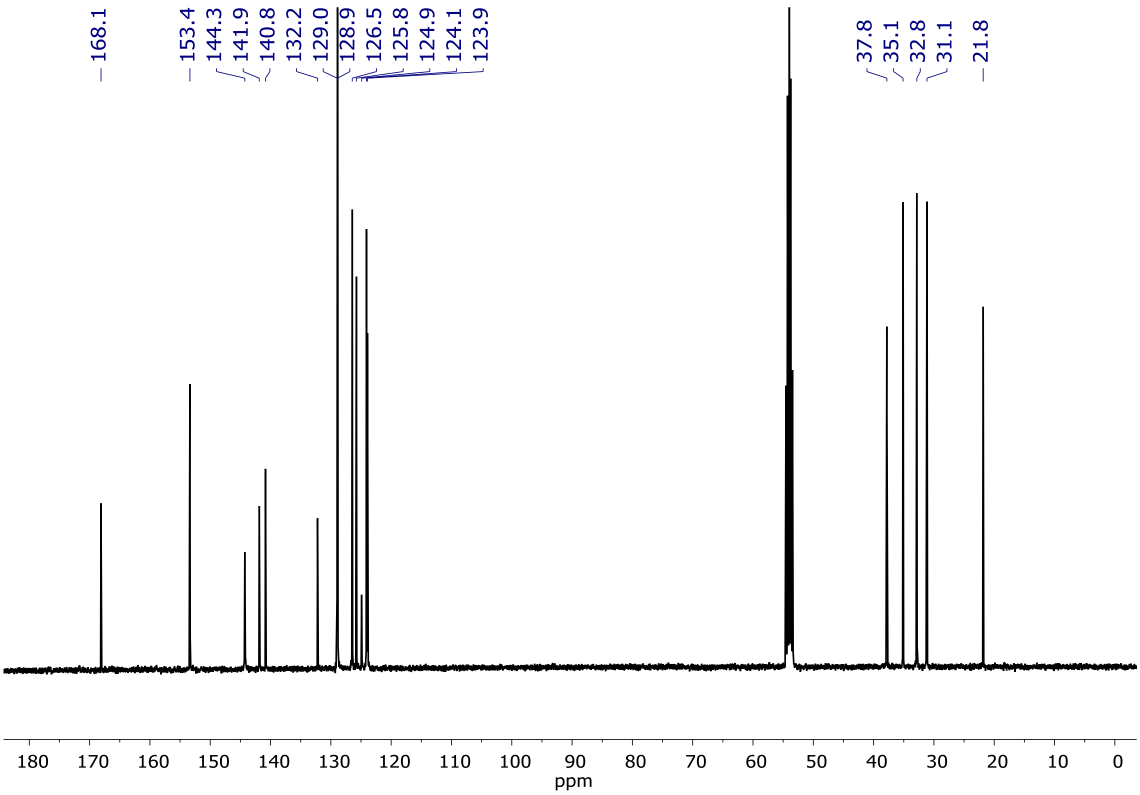

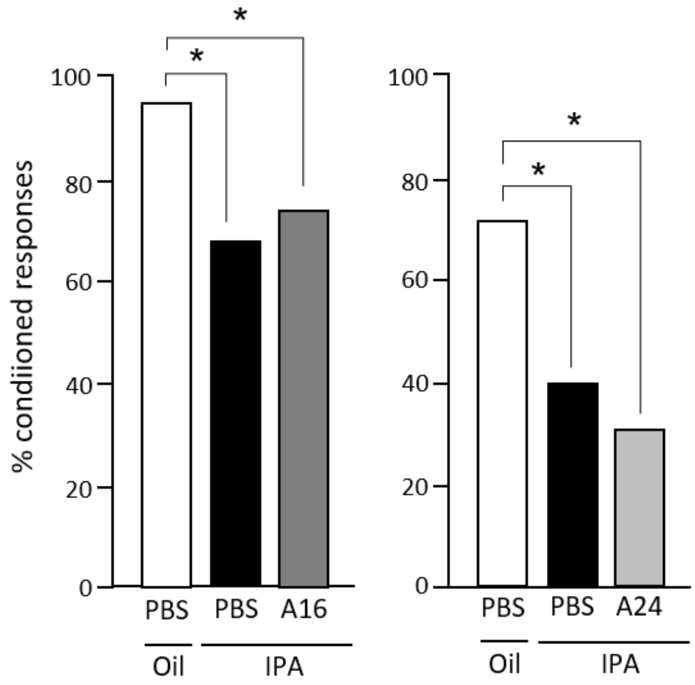

Supplementary Fig.1: Absence of effect of A16 and A23 on stressed bees. Proportions of individuals showing a conditioned response to the odorant paired with sucrose, at the end of the conditioning session (third trial). Bees were injected either with PBS, A16 or A23 (10^{-4} M), then exposed to IPA or paraffin oil only (negative controls). Exposure to IPA significantly reduced learning performance in PBS-injected bees, but also in those injected with high doses of A16 or A23, which had thus no detectable effect on the impact of stress on learning performance. *: $p<0.025$.

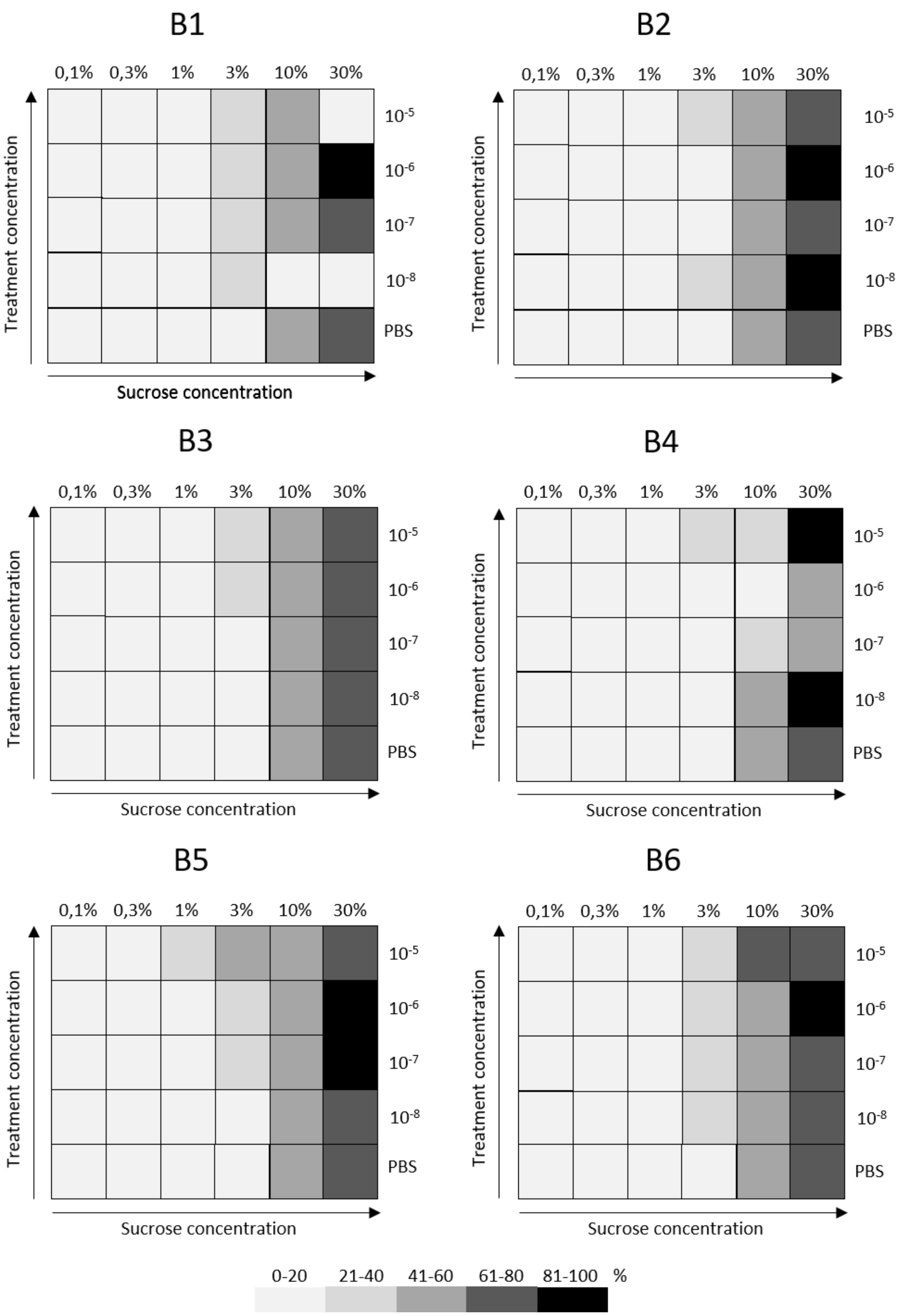

Supplementary Fig.2: Responses to increasing sucrose concentrations in stressed bees treated with the B series. For each treatment (molecule/dose), the percentage of responses to a given sucrose concentration is colour-coded. While overall, bees increasingly responded to increasing sucrose concentrations, this trend was differently modulated by B molecules. The pooled values of all PBS-treated bees were used to provide an identical reference in all matrices. (PBS: n=114; B compounds: 28-41/group) (2 replicates).

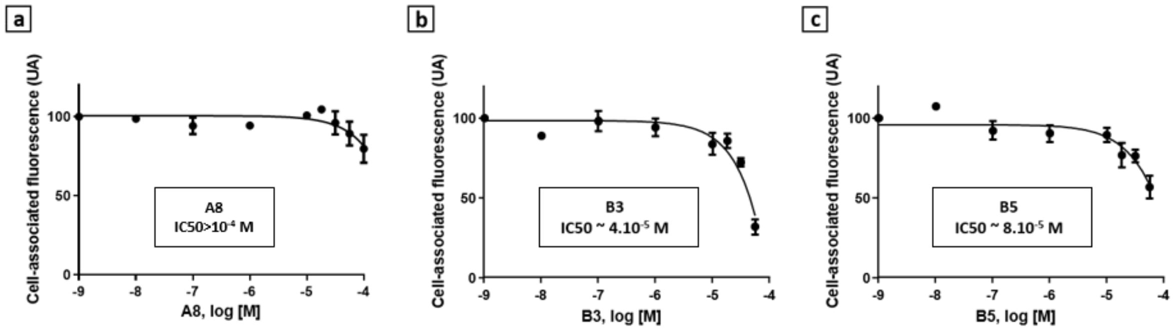

Supplementary Fig.3: *In vitro* competitive binding assays for the B3 and B5 molecules. Competition curves and obtained IC_{50} values for the B3 and B5 molecules (5 replicates), as compared to that of A8.
